# Rare‐Earth‐ and Uranium‐Mesoionic Carbenes: A New Class of f‐Block Carbene Complex Derived from an N‐Heterocyclic Olefin

**DOI:** 10.1002/anie.201706546

**Published:** 2017-08-09

**Authors:** John A. Seed, Matthew Gregson, Floriana Tuna, Nicholas F. Chilton, Ashley J. Wooles, Eric J. L. McInnes, Stephen T. Liddle

**Affiliations:** ^1^ School of Chemistry The University of Manchester Oxford Road Manchester M13 9PL UK; ^2^ School of Chemistry and Photon Science Institute The University of Manchester Oxford Road Manchester M13 9PL UK

**Keywords:** density functional theory, mesoionic carbenes, N-heterocyclic olefins, rare earth elements, uranium

## Abstract

Neutral mesoionic carbenes (MICs) have emerged as an important class of carbene, however they are found in the free form or ligated to only a few d‐block ions. Unprecedented f‐block MIC complexes [M(N′′)_3_{CN(Me)C(Me)N(Me)CH}] (M=U, Y, La, Nd; N′′=N(SiMe_3_)_2_) are reported. These complexes were prepared by a formal 1,4‐proton migration reaction when the metal triamides [M(N′′)_3_] were treated with the N‐heterocyclic olefin H_2_C=C(NMeCH)_2_, which constitutes a new, general way to prepare MIC complexes. Quantum chemical calculations on the 5f^3^ uranium(III) complex suggest the presence of a U=C donor‐acceptor bond, composed of a MIC→U σ‐component and a U(5f)→MIC(2p) π‐back‐bond, but for the d^0^f^0^ Y and La and 4f^3^ Nd congeners only MIC→M σ‐bonding is found. Considering the generally negligible π‐acidity of MICs, this is surprising and highlights that greater consideration should possibly be given to recognizing MICs as potential π‐acid ligands when coordinated to strongly reducing metals.

Over the past three decades the field of stable singlet N‐heterocyclic carbenes (NHCs, **I**, Scheme 1) has become a burgeoning area.^1^ Within that time, a variety of experimentally viable classes of carbenes related to **I** have emerged, including anionic‐NHCs (**II**),^2^ cyclic alkylaminocarbenes (CAAC, **III**),^3^ and various charge‐neutral mesoionic carbenes (MIC, **IV**–**VI**),^4^ Scheme 1, where for the latter no reasonable canonical resonance forms can be drawn without assigning additional formal charges. A growing number of MICs of type **IV** are known, but it is notable that all examples to date pertain to either the free carbene, or were formed at and remain coordinated to surprisingly few transition metal ions,^4^ which contrasts to NHCs that have been coordinated to the majority of metals in the periodic table.^1^ Where the bonding of these MICs to metals is concerned, complexes are usually considered to have strong MIC→metal σ‐donation. Given that strong σ‐donation, it is surprising that MIC complexes are limited to even only a few transition metals, but this may reflect the limited range of methodologies to deliberately prepare metal‐MIC complexes. Interestingly, any π‐bonding components of metal‐MIC bonds are, unlike NHCs, rarely explicitly considered.^5^ This is likely because MICs are anticipated to have at best weak π‐acceptor character since the carbene is strongly stabilized by *N*‐lone pair and vinyl groups, as evidenced by computational comparisons of different classes of carbenes.^6^

**Scheme 1:**
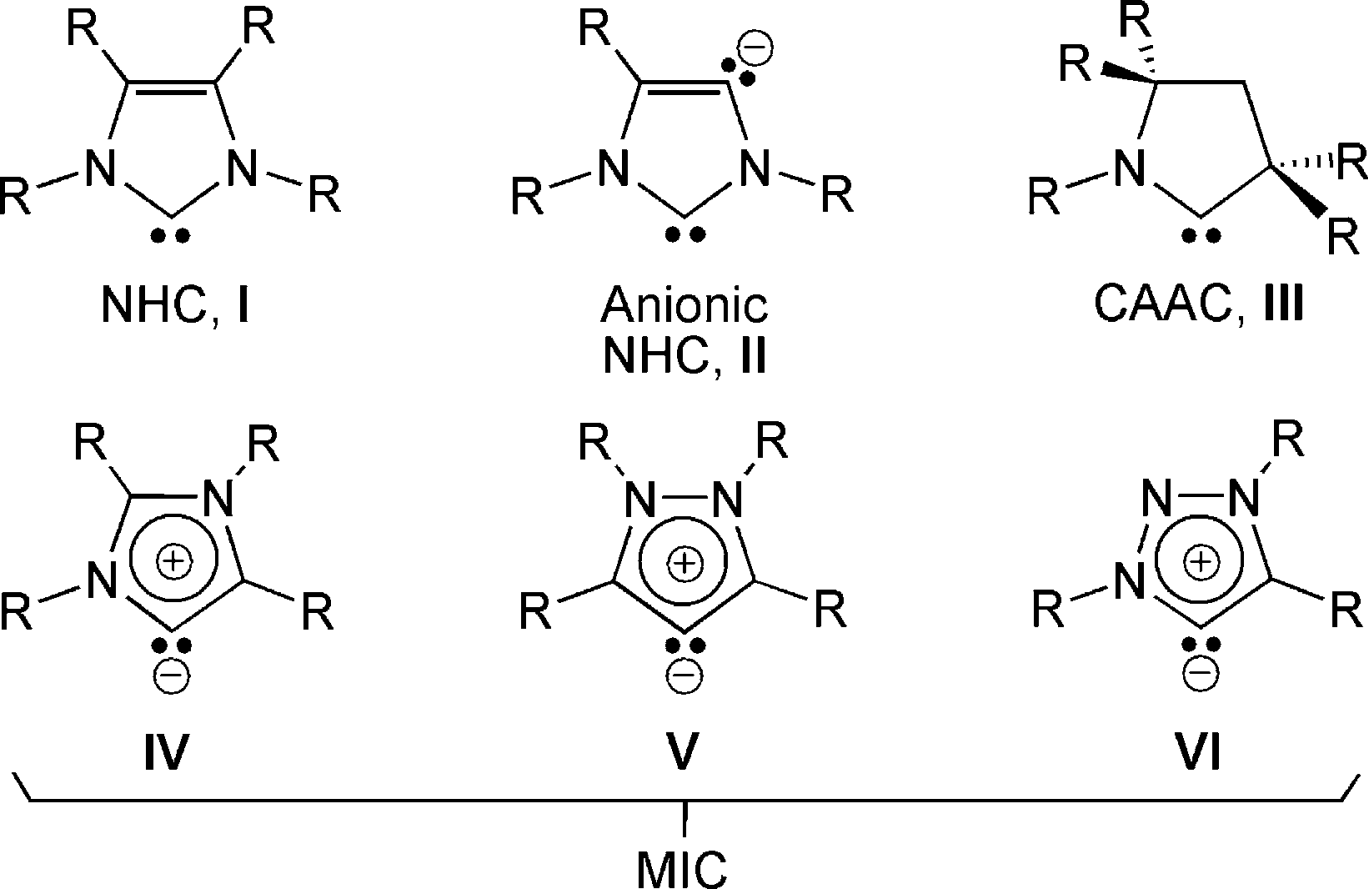
Prominent classes of NHC‐type 5‐membered‐ring carbenes.

We report herein the synthesis and characterization of rare‐earth–MIC and uranium–MIC complexes, which are the first f‐block‐MIC complexes so by definition a new class of f‐block‐carbene complex.^7^ The complexes reported herein were prepared by the formal 1,4‐proton migration of an N‐heterocyclic olefin (NHO) that represents a new, general method by which to prepare MIC complexes. Interestingly, quantum chemical calculations suggest that the 5f^3^ uranium(III) ion engages in a weak π‐back‐bond to the MIC utilizing a 5f electron, whereas the corresponding d^0^f^0^ yttrium(III) and lanthanum(III), and 4f^3^ neodymium(III) benchmarks do not. Considering the generally negligible π‐acidity of MICs,^6^ this result for uranium is surprising, and highlights that perhaps greater consideration should be given to more widely recognizing MICs as potential π‐acid ligands when coordinated to sufficiently reducing metals.

As part of our continued studies of f‐element–carbon multiple bonding,^8^ we examined the reactivity of the uranium(III)–triamide complex [U(N′′)_3_] (**1U**, N′′=N(SiMe_3_)_2_)^9^ with the NHO H_2_C=C(NMeCH)_2_ (**2**).^10^ We postulated that an adduct similar to [Nd(N′′)_3_{H_2_C=C(NMeCMe)_2_}] could form,^11^ which in one resonance form can be represented as a NHC‐protected methylidene, but also that as well as being nucleophilic the methylene groups of NHOs are basic by virtue of the dipolar resonance form H_2_C^−^‐C^+^(NRCH)_2_.^12^ Given the existence of MICs, we considered whether **2** could be converted by transfer of an olefinic hydrogen atom to the methylene group in a formal 1,4‐proton shift to give its MIC form with concomitant metal stabilization.

Experimentally, we find that reaction of **1U** with **2** gives [U(N′′)_3_{CN(Me)C(Me)N(Me)CH}] (**3U**), Scheme 2.^13^ The crystalline yield of **3U** (27 %) is low, but is due to the high solubility of this complex since inspection of the mother liquor by NMR spectroscopy shows that **3U** is the major product. How **3U** forms is unclear, as no intermediates could be observed in low‐temperature NMR studies. A formal, concerted 1,4‐proton shift might be promoted by **1U**, or more likely **1U** might C4‐deprotonate **2** and the resulting N′′H could reprotonate the putative [U(N′′)_2_{CN(Me)C(CH_2_)N(Me)CH}] at the basic methylene group to re‐establish the third uranium‐amide bond and restore overall charge neutrality to the MIC. This is credible because reactions conducted in D_6_‐benzene show no evidence for D‐incorporation into **3U**. To test the generality of this new reaction, we tested the reactivity of [M(N′′)_3_] (M=Y, **1Y**; La, **1La**; Nd, **1Nd**) with **2** since the first two are closed‐shell d^0^f^0^ analogues and the latter is a 4f^3^ congener to 5f^3^ **3U**. Remarkably, all consistently give the MIC complexes [M(N′′)_3_{CN(Me)C(Me)N(Me)CH}] (M=Y, **3Y**; La, **3La**; Nd, **3Nd**), isolated in crystalline yields of 23–30 %, Scheme 2.^13^

**Scheme 2:**
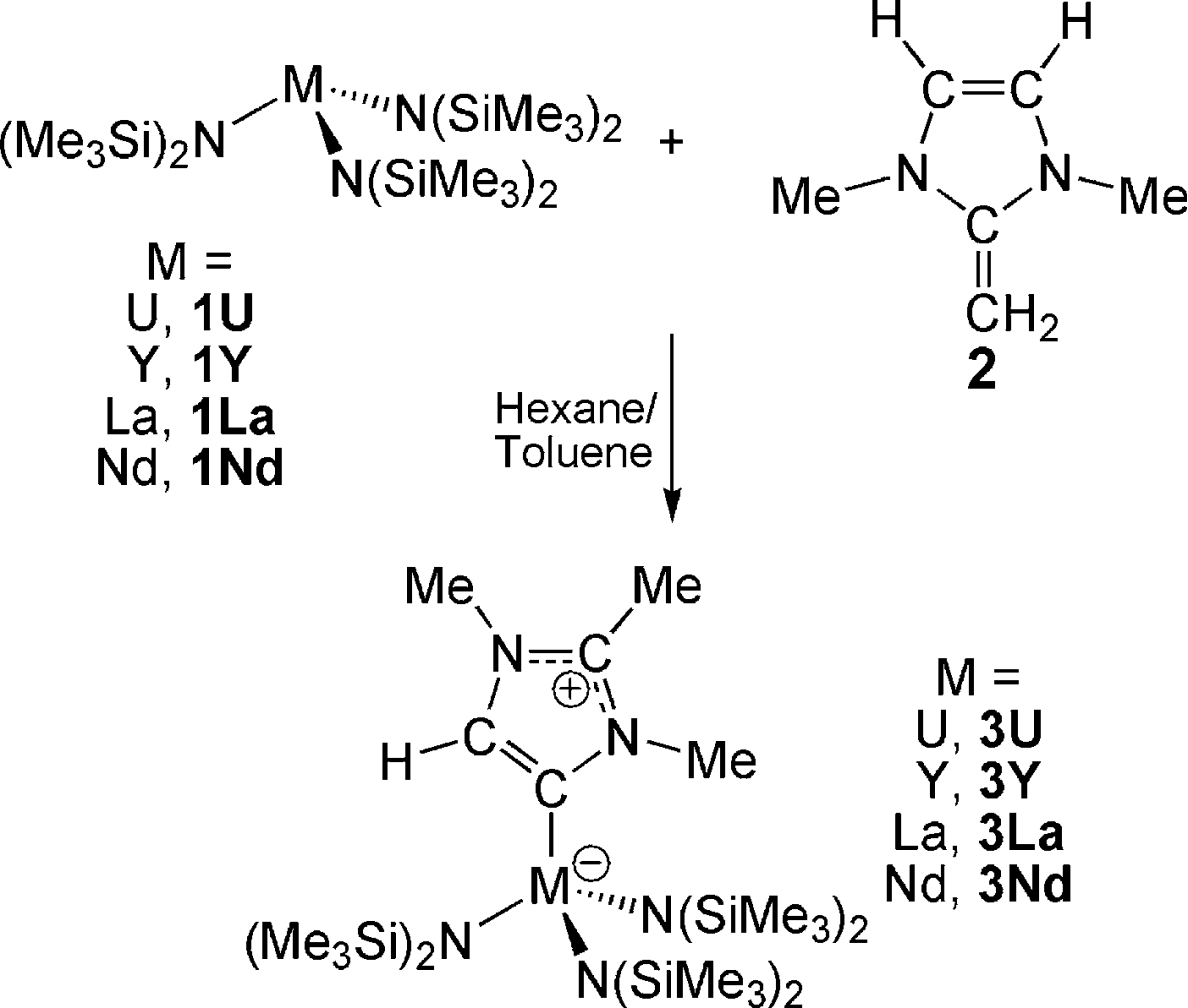
Synthesis of the f‐block‐mesoionic carbene complexes **3M** (M=U, Y, La, Nd) from the N‐heterocyclic olefin **2** and the metal triamides **1M**.

The molecular structures of **3U**, **3Y**, **3La**, and **3Nd** were determined by single‐crystal X‐ray diffraction (Figure 1).^14^ In multiple crystals, the MIC is disordered, but modeled reliably, over three positions for **3U**, two for **3La** and **3Nd**, but it is ordered for **1Y**, which can be related to the size of the metal and pocket that the MIC sits in. There are no other f‐block‐MICs for comparison, and few f‐block‐NHCs have a sterically comparable profile to **3U**, **3Y**, **3La**, and **3Nd**, but for example the U−C_carbene_ distances in **3U** (2.576(12)–2.598(11) Å), can be compared to the longer U−C_carbene_ distance in [U(N′′)_3_{C(NMeCMe)_2_}] [2.672(5) Å],^15^ and the U−C_ylide_ bond length of 2.686(6) Å in [U(N′′)_3_(H_2_CPPh_3_)].^16^ Likewise, the M–MIC distances in **3Y** (2.495(7) Å), **3La** (2.675(14)/2.699(5) Å), and **3Nd** (2.614(12)/2.620(11) Å) are consistently short when compared to respective NHC congeners.^17^ The MIC ring bond distances are intermediate to single and double bonds, suggesting delocalization. The U−N distances in **3U** span 2.359(7)–2.381(7) Å, which suggests the uranium ion retains a+3 oxidation state. For comparison, U−N distances in **1U**,^9^ [U(N′′)_3_{C(NMeCMe)_2_}],^15^ and [U(N′′)_3_(H_2_CPPh_3_)]^16^ average 2.320(4), 2.362(3), and 2.364(9) Å, respectively, whereas for [U(N′′)_3_(I)]^18^ they are 2.238(4) Å. The M−N distances in **3Y**, **3La**, and **3Nd** are also consistent with their M^III^ natures.

**Figure 1:**
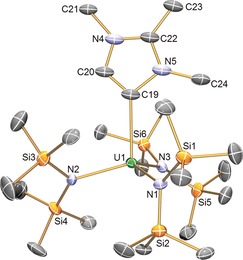
Molecular structure of **3U** at 150 K with ellipsoids set to 50 % probability. Hydrogen atoms and disorder components are omitted for clarity. The structures of **3Y**, **3La**, and **3Nd** are very similar.^14^

NMR spectra of **3U**, **3Y**, **3La**, and **3Nd** are consistent with their M^III^ formulations,^19^ and a doublet at 173 ppm in the ^13^C{^1^H} spectrum of **3Y** (*J*_YC_=56 Hz, ^89^Y; *I*=1/2 , 100 %)^20^ and absence of any carbene resonance for **3La** due to the quadrupolar lanthanum (^139^La; *I*=7/2, 99.9 %) suggests that the MICs remain ligated in solution for all complexes.

The UV/Vis/NIR spectrum of **3U** exhibits broad absorptions in the region 14 800–21 000 cm^−1^ (*ϵ*=530–870 L mol^−1^ cm^−1^) characteristic of 5f^3^6d^0^ → 5f^2^6d^1^ transitions of uranium(III) along with weaker (*ϵ*<180 L mol^−1^ cm^−1^) absorptions in the NIR region are observed.8j, 9b This is similar to that of **1U**,^9^ but distinct to [U(N′′)_3_(I)].^18^

The uranium(III) assignment of **3U** was further confirmed by SQUID magnetometry and EPR spectroscopy (Figure 2). Powdered **3U** exhibits a magnetic moment of 2.82 μ_B_ at 300 K (3.31 μ_B_ in solution at 298 K), Figure 2 (Left). This is lower than the theoretical magnetic moment of 3.62 μ_B_ for a uranium(III) ion (^4^I_9/2_ ground spin–orbit multiplet, *g_J_*=8/11), owing to crystal/ligand field splitting, and is generally in‐line with uranium(III) magnetic moments.^21^ Characteristic of uranium(III), the magnetic moment of **3** decreases slowly across the entire temperature range, reaching 2.12 μ_B_ at 2 K. Furthermore, low‐temperature magnetization data saturate at moderate magnetic fields, consistent with the Kramers nature of the uranium(III) ion, Figure 2 (middle). For comparison, we re‐measured data for **1U**,^9^, ^15^, ^21^ and [U(N′′)_3_(I)],^18^ giving data consistent with literature values. Importantly, **1U** has a similar low‐temperature magnetization profile to **3U**, while that of the non‐Kramers [U(N′′)_3_(I)] fails to saturate up to 7 T. Further support for the uranium(III) oxidation state of **3U** comes from low temperature (20 K) EPR spectroscopy at 9.5 GHz (Figure 2, right), where a typical uranium(III) spectrum^22^ is observed from a powdered sample with effective *g*‐values of *g*=4.65, 1.33, and 0.89 arising from the ground Kramers doublet. The non‐Kramers uranium(IV) ion would be expected to be EPR‐silent under these conditions. An isolated Kramers doublet with these *g*‐values corresponds to a magnetic moment of 2.46 μ_B_, in fair agreement with the experimental magnetic moment of 2.27 μ_B_ observed at 20 K. Furthermore, these *g*‐values and magnetic moments are in reasonable agreement with those determined with CASSCF‐SO calculations,^13^ which predict *g*=4.3, 2.4, and 0.7 for the ground Kramers doublet (weighted for the crystal structure MIC disorder; compare to calculated *g*_∥_=0.6 and *g*_⊥_=3.3 for **1U**) and magnetic moments of 2.48 and 3.28 μ_B_ at 2 and 298 K (Figure 2, left, inset).

**Figure 2:**
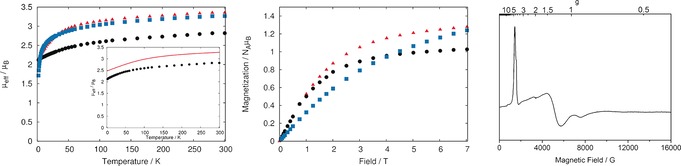
Left: Temperature‐dependent SQUID data for powdered samples of the uranium(III) complexes **1U** (red ▴) and **3U** (black •), and the uranium(IV) complex [U(N′′)_3_(I)] (blue ▪) recorded in a 0.1 T magnetic field over the temperature range 2 to 298 K; inset: CASSCF‐SO calculated (red —) and experimental (black —) magnetic data for **3U**. Middle: Field dependent SQUID data for powdered samples of the uranium(III) complexes **1U** (red ▴) and **3U** (black •), and the uranium(IV) complex [U(N′′)_3_(I)] (blue ▪) recorded at 1.8 K over the magnetic field range 0 to 7 T. Right: X‐band (ca. 9.5 GHz) EPR spectrum of a powdered sample of **3U** at 20 K.

To probe the nature of the metal–carbene linkages in **3U**, **3Y**, **3La**, and **3Nd**, we calculated their electronic structures in detail, noting that d^0^f^0^ **3Y** and **3La** and 4f^3^ **3Nd** represent closed‐shell and f^*n*^‐analogues for benchmarking purposes, respectively. The Kohn–Sham molecular orbitals of **3U** reveal a U=C donor–acceptor interaction, where resonance forms **3U‐a** and **3U‐b** can be invoked, Scheme 3. The MIC→U two‐electron σ‐donation is represented by HOMO−16, and HOMO−1 reveals U→MIC one‐electron π‐back‐donation from a uranium 5f‐orbital to an empty carbene p‐character orbital that is generated in resonance form **3U‐b**. The HOMO and HOMO−2 account for the remaining two 5f electrons of uranium(III). The donor–acceptor character of **3U** stands in contrast to **3Y**, **3La**, and **3Nd** where, as expected, only the σ‐component to the bonding is found in the Kohn–Sham orbitals and thus only resonance form **3M‐a** is invoked (Scheme 3). Complexes **3Y** and **3La** as d^0^f^0^ complexes would certainly not be expected to exhibit such donor–acceptor character and indeed the molecular orbital that would constitute a M→MIC back‐bond is in both cases the LUMO+1 orbitals with carbene 2p and 4d (Y) and 5d/4f (La) character that sit about 2.5 and about 3.6 eV above the respective HOMO orbitals. For **3Nd**, HOMO−2 to HOMO are the 4f electrons, then LUMO to LUMO+4 are dominated by virtual 4f/amide combinations before the relevant Nd→MIC interaction (5d/2p) is found in LUMO+5, some 2.5 eV above the HOMO. Thus, **3Y** and **3La** do not have the requisite electrons to back‐bond, and **3Nd** has the electrons but they are energetically incompatible with back‐bonding to the MIC.

**Scheme 3:**
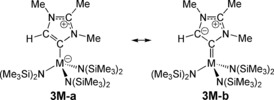
Two of the principal resonance forms for the MIC complex **3U**.

The computed MDC‐q charges and Nalewajski–Mrozek bond orders of **3U**, **3Y**, **3La**, and **3Nd** are instructive and fall into two distinct groups of **3U** and **3Y**/**3La**/**3Nd**. Specifically, the U, C_carbene_, α‐C, and α‐N charges are 1.8, −0.81, −0.05, and −0.31 with U=C, C=C, and C−N bond orders of 1.1, 1.64, and 1.22, respectively. Those of **3Y**, **3La**, and **3Nd** are remarkably invariant with av. M, C_carbene_, α‐C, and α‐N charges of 1.35, −0.6, −0.04, and −0.29 and M−C, C=C, and C−N bond orders of 0.6, 1.73, and 1.26, respectively. If there is no back‐bonding, the carbene should show strong stabilizing interactions with the α‐C and ‐N atoms, and the metal and carbene should have low positive and negative charges, respectively. Conversely, if back‐bonding operates in addition to the σ‐donation then the metal and carbene should have higher positive and negative charges, respectively, reflecting the transfer of electron density back from the metal to carbene, and the carbene should consequently have weaker bonding interactions with the α‐C and ‐N atoms. This is exactly the situation that is suggested by the calculations, consistent with the Kohn–Sham descriptions. We note that the metal–carbene bond order in **3U** is nearly twice that of **3Y**, **3La**, and **3Nd**, and, recalling that the U→MIC π‐back‐bond involves a singly occupied 5f‐orbital, that it is greater than one suggests the presence of a two‐fold bonding interaction where each component is polarized and of sub‐integer bond order. Considering the generally accepted negligible π‐acidity of MICs the donor–acceptor bond in **3U** is notable, and is also remarkably similar to the donor–acceptor interaction found computationally in [U(N′′)_3_{C(NMeCMe)_2_}].^15^ However, we note that the back‐bond must be weak because we could not freeze‐out rotation of the MIC by the solvent low‐temperature limit (−80 °C) in NMR studies.

NBO analysis of **3U** (Figure 3) is also consistent with a U=C donor–acceptor interaction. The MIC→U σ‐donation is returned as essentially electrostatic and so is predominantly carbon‐based. However, the U→MIC π‐back‐donation is found to contain 75 % uranium and 25 % carbene character. The carbene acceptor orbital is a pure 2p orbital, whereas the uranium donor orbital is 90 % 5f and 10 % 6d character. As expected, only electrostatic NBO MIC→M interactions are found for **3Y**, **3La**, and **3Nd**.

**Figure 3:**
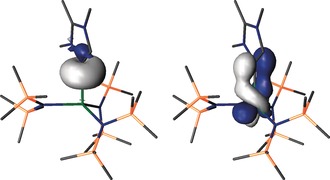
Natural bond orbitals calculated for **3U** with hydrogen atoms omitted for clarity. Left: the carbene to uranium two‐electron σ‐bond component. Right: the uranium to carbene single‐electron π‐back‐bonding interaction.

Along with the orbital‐based perspectives of DFT and NBO analyses we probed the topological electron density description of the M−C bonds in **3U**, **3Y**, **3La**, and **3Nd**. The calculations reveal M−C 3,−1 critical points. The *ρ*(*r*)_MC_ values are similar for all complexes (0.08–0.12) suggesting polar interactions, since covalent bonds tend to have *ρ*(*r*) >0.1, but we note that **3U** has the highest *ρ*(*r*)_MC_ value. Most importantly, however, the calculated ellipticity parameters *ϵ*(r)_MC_ are 0.03, 0.06, and 0.09 for **3Y**, **3La**, and **3Nd**, respectively, but for **3U** the *ϵ*(*r*)_UC_ value is 0.36. This supports the notion of a polarized two‐fold U=C bonding interaction in **3U** because a single σ bond or triple σ–π–π bond present symmetrical electron density distributions around the inter‐nuclear axes (*ϵ*(*r*)≈0) whereas σ–π double bonds are asymmetric (*ϵ*(*r*)>0). For comparison, calculated C−C *ϵ*(*r*)_CC_ values in ethane (H_3_C−CH_3_), benzene (C_6_H_6_), ethylene (H_2_C=CH_2_), and acetylene (HC≡CH) are 0.0, 0.23, 0.45, and 0.0, respectively.^23^

Inspection of the CASSCF‐SO *m_J_* manifolds of **1U** and **3U** reveals that there is only a small change in the energies of the three lowest doublets, whilst the two highest energy states are suppressed by about 400 cm^−1^ in **3U** compared to **1U**. We also observe a clear change in the *g*‐values of the ground doublet, reflecting the departure from axial symmetry in **3U**, as confirmed experimentally. These modest changes reflect the coordination of the MIC and also the electronic partial‐cancellation effects of the U=C donor–acceptor interaction, analogous to donor–acceptor net‐cancellation effects on the CO stretching frequency of thorium carbonyls.^24^

To conclude, we have prepared the first examples of f‐block‐MICs, which thus represent a new class of f‐block carbene. These complexes were prepared by a formal 1,4‐proton migration of an NHO, which therefore represents a new, general way to prepare MIC complexes. Quantum chemical calculations suggest that in addition to a MIC→U σ‐donation there is a weak U(5f)→MIC(2p) π back‐bond; although resonance form **3U‐a** most likely dominates, resonance form **3U‐b** is non‐negligible. As expected **3Y** and **3La** exhibit no back‐bonding due to their d^0^f^0^ natures, and **3Nd** though being 4f^3^ also does not back‐bond as its valence 4f electrons are energetically incompatible to do this. The donor–acceptor character in **3U** is reminiscent of d‐block‐carbonyl and Fischer carbene bonding, though the π‐back‐bond is weak. Considering the generally at best weak π‐acidity of MICs, the computational finding of U(5f)→MIC(2p) π‐back‐bond is surprising and highlights that perhaps greater consideration should be given to more widely acknowledging MICs as potential π‐acid ligands when coordinated to sufficiently reducing metals.

## Conflict of interest

The authors declare no conflict of interest.

## Supporting information

As a service to our authors and readers, this journal provides supporting information supplied by the authors. Such materials are peer reviewed and may be re‐organized for online delivery, but are not copy‐edited or typeset. Technical support issues arising from supporting information (other than missing files) should be addressed to the authors.

Supplementary
