# Supplementary material for: Rare‐Earth‐ and Uranium‐Mesoionic Carbenes: A New Class of f‐Block Carbene Complex Derived from an N‐Heterocyclic Olefin
Source: Angew Chem Int Ed Engl. 2017 Aug 9;56(38):11534–8. doi: 10.1002/anie.201706546 (PMC5601227; doi:10.1002/anie.201706546)
Supplement: Supplementary file 1 — Supplementary [file ANIE-56-11534-s001.pdf]

Angewandte Chemie
Eine Zeitschrift der Gesellschaft Deutscher Chemiker

Supporting Information

Rare Earth- and Uranium-Mesoionic Carbenes: A New Class of f-Block Carbene Complex Derived from an N-Heterocyclic Olefin

*John A. Seed, Matthew Gregson, Floriana Tuna, Nicholas F. Chilton, Ashley J. Wooles, Eric J. L. McInnes, and Stephen T. Liddle**

anie_201706546_sm_miscellaneous_information.pdf

Experimental

General

All manipulations were carried out under an inert atmosphere of dry N_2 using Schlenk techniques or an MBraun UniLab glovebox. All glassware was silylated and dried prior to use. Solvents were dried by passage through activated alumina towers and degassed prior to use. All solvents were stored over potassium mirrors, except for ethers that were stored over activated 4Å molecular sieves. Deuterated solvent was distilled from a potassium mirror and degassed by three freeze-pump-thaw cycles and stored under N_2. The compounds [U(N")$_3$] (**1U**), 1,3-dimethyl-2-methylene imidazoline (**2**), and [U(N")$_3$(I)] (**4**) were synthesised according to published procedures.[1-3] The compounds [M(N")$_3$] [M = Y (**1Y**), La (**1La**), Nd (**1Nd**)] were purchased from Sigma Aldrich and sublimed prior to use.

^{1}H, ^{13}C{^{1}H}, and ^{29}Si{^{1}H} NMR spectra were recorded on a Bruker DPX400 spectrometer operating at 400.13, 100.61, and 79.49 MHz, respectively. Chemical shifts (δ) are quoted in ppm (parts per million) and are relative to external TMS (tetramethylsilane). Infra-red (IR) spectra were obtained using a Bruker Alpha Platinum-ATR FTIR spectrometer. UV/Vis/NIR spectra were recorded on a Perkin Elmer Lambda 750 spectrometer where data were collected in 1mm path length cuvettes and were run versus the appropriate reference solvent. Static variable-temperature magnetic moment data were recorded in an applied dc field of 0.1 T on a Quantum Design MPMS XL7 superconducting quantum interference device (SQUID) magnetometer using doubly recrystallised powdered samples. Samples were carefully checked for purity and data reproducibility between several independently prepared batches for each compound examined. Data were measured on cooling and warming (average values presented), and samples were immobilised in an eicosane matrix to prevent sample reorientation during measurements. Because samples could not be finely ground (see below), samples for SQUID measurements were rapidly crash-crystallised to give crops of a fine/powdery consistency to avoid the need to grind them. Diamagnetic

corrections of were applied using tabulated Pascal constants and measurements were corrected for the effect of the blank sample holders (flame sealed Wilmad NMR tube and straw) and eicosane matrix. EPR spectra recorded on samples prepared likewise to the SQUID samples were measured on a Bruker Elexsys E500 spectrometer. Samples of **3U** show clear evidence of anisotropy as evidenced by the broad linewidths. After extensive experiments it was determined that 20 K was the best measurement temperature, in terms of stability of the measurement temperature and also spectral resolution. Elemental microanalyses were carried out by Mr Martin Jennings and Mrs Anne Davies at The University of Manchester. Considerable issues were consistently encountered obtaining CHN data for **3U**, **3Y**, **3La**, and **3Nd**. Specifically, despite loading pristine crystalline materials into the elemental analysis combustion boats, in common with other organosilicon-rich compounds,[4] silicon-carbide formation precluded complete combustion and the instrument temperatures could not be raised high enough to overcome this problem. Combustion aids did not ameliorate this situation. This issue was compounded by the fact that crystalline samples of **3U**, **3Y**, **3La**, and **3Nd** cannot be finely ground to aid combustion because even with stringent precautions the mechanical action of grinding degrades crystalline samples of **3U**, **3Y**, **3La**, and **3Nd** to unknown oily yellow materials; note also that minor impurities in NMR spectra result from slow decomposition of **3U**, **3Y**, **3La**, and **3Nd** over time. Nevertheless, the characterisation data when taken together support the proposed formulation of **3U**, **3Y**, **3La**, and **3Nd**.

Preparation of 3U

A solution of **2** (0.11 g, 1.00 mmol) in toluene (10 ml) was added dropwise to a pre-cooled (–20 °C) solution of **1U** (0.72 g, 1.00 mmol) in hexane (30 ml). The reaction mixture was stirred for 12 hours at room temperature. The solvent was then removed *in vacuo* to afford a dark purple residue. The resulting solid was extracted into hexane (10 ml) and stored at 2 °C to afford **3** as purple crystals. Isolated crystalline yield: 0.22 g, 27%. Anal. Calcd for $C_{24}H_{64}N_5Si_6U$: C, 34.79; H, 7.78; N, 8.45%. Found: C, 33.05; H, 7.58; N, 8.13%. Consistently low C-values were repeatedly obtained in elemental analyses. ^{1}H NMR (C_6D_6, 298 K): δ 5.69 (br, s, 3H, $C(CH_3)$), –6.04 (br, s, 54H,

$Si(CH_3)_3$), –7.24 (s, 3H, $N(CH_3)$), –7.87 (br, s, 3H, $N(CH_3)$), –31.38 (br, s, 1H, C=C(H)). $^{13}C\{^1H\}$ NMR ($C_6D_5CD_3$, 298K): δ –98.36 ($Si(CH_3)_3$) No other ^{13}C resonances for **3U** could be observed. $^{29}Si\{^1H\}$ NMR (C_6D_6, 298 K): δ –118.03. ATR-IR *ν*/cm^{-1}: 2946 (m), 2895 (w), 1576 (w), 1555 (w), 1518 (w, br), 1411 (w, br), 1238 (s), 952 (s), 818 (s), 767 (s), 661 (s), 599 (s). μ_{eff} (Evans method, C_6D_6, 298 K): 3.31 μ_B.

Preparation of 3Y

A solution of **2** (0.11 g, 1.00 mmol) in toluene (10 ml) was added dropwise to a pre-cooled (–20 °C) solution of **1Y** (0.57 g, 1.00 mmol) in hexane (30 ml). The reaction mixture was stirred for 48 hours at room temperature. The volume was then reduced by half *in vacuo* and stored at –30 °C to afford **3Y** as colourless crystals. Isolated crystalline yield: 0.20 g, 29%. Anal. Calcd for $C_{24}H_{64}N_5Si_6Y$: C, 42.40; H, 9.50; N, 10.31%. Found: C, 39.31; H, 8.94; N, 10.42%. ^1H NMR (C_6D_6, 298 K): δ 6.51 (s, 1H, C=C(H)), 3.52 (s, 3H, $C(CH_3)$), 1.97 (s, 3H, $N(CH_3)$), 1.04 (s, 3H, $N(CH_3)$), 0.52 (s, 54H, $Si(CH_3)_3$). $^{13}C\{^1H\}$ NMR (C_6D_6, 298K): δ 172.75 (d, J_{YC} 56.2 Hz, $C_{carbene}$), 139.46 (C=C(H)), 38.30 ($N(CH_3)$), 32.17 ($N(CH_3)$), 7.14 ($C(CH_3)$), 6.37 ($Si(CH_3)_3$). $^{29}Si\{^1H\}$ NMR (C_6D_6, 298 K): δ –10.92. ATR-IR ν/cm^{-1}: 2944 (m), 2893 (w), 1576 (w), 1411 (w, br), 1237 (s), 941 (s), 862 (w), 818 (s), 770 (m), 748 (m), 659 (s), 607 (s).

Preparation of 3La

A solution of **2** (0.11 g, 1.00 mmol) in toluene (10 ml) was added dropwise to a pre-cooled (–20 °C) solution of **1La** (0.62 g, 1.00 mmol) in hexane (30 ml). The reaction mixture was stirred for 48 hours at room temperature. The volume was then reduced by half *in vacuo* and stored at –30 °C to afford **3La** as orange crystals. Isolated crystalline yield: 0.17 g, 23%. Anal. Calcd for $C_{24}H_{64}N_5Si_6La$: C, 39.49; H, 8.84; N, 9.60%. Found: C, 38.78; H, 8.77; N, 9.41%. ^1H NMR (C_6D_6, 298 K): δ 6.43 (s, 1H, C=C(H)), 3.43 (s, 3H, $C(CH_3)$), 2.01 (s, 3H, $N(CH_3)$), 0.93 (s, 3H, $N(CH_3)$), 0.52 (s, 54H, $Si(CH_3)_3$). $^{13}C\{^1H\}$ NMR (C_6D_6, 298K): δ 139.39 (C=C(H)), 38.00 ($N(CH_3)$), 31.87

(N(CH_3)), 7.48 (C(CH_3)), 5.41 (Si(CH_3)$_3$). The carbene resonance was not observed, which we attribute to rapid relaxation due to coordination to the quadrupolar ^{139}La (99.91% $I = 7/2$) nucleus in solution. ^{29}Si{^{1}H} NMR (C_6D_6, 298 K): δ –13.37. ATR-IR ν/cm^{-1}: 2945 (m), 2894 (w), 1575 (w), 1411 (w, br), 1239 (s), 961 (s), 862 (m), 814 (s), 766 (m), 661 (s), 594 (s).

Preparation of 3Nd

A solution of **2** (0.11 g, 1.00 mmol) in toluene (10 ml) was added dropwise to a pre-cooled (–20 °C) solution of **1Nd** (0.63 g, 1.00 mmol) in hexane (30 ml). The reaction mixture was stirred for 48 hours at room temperature. The volume was then reduced by half *in vacuo* and stored at –30 °C to afford **3Nd** as green crystals. Isolated crystalline yield: 0.17 g, 23%. Anal. Calcd for $C_{24}H_{64}N_5Si_6Nd$: C, 39.20; H, 8.78; N, 9.53%. Found: C, 38.95; H, 8.91; N, 9.66%. ^{1}H NMR (C_6D_6, 298 K): δ 15.52 (br, s, 3H, C(CH_3)), –1.15 (br, s, 54H, Si(CH_3)$_3$), –2.24 (s, 3H, N(CH_3)), –6.06 (br, s, 3H, N(CH_3)), –29.80 (br, s, 1H, C=C(H)). ^{29}Si{^{1}H} NMR (C_6D_6, 298 K): δ –47.11. ATR-IR ν/cm^{-1}: 2945 (m), 2893 (w), 1573 (w), 1407 (w, br), 1239 (s), 955 (s), 861 (m), 815 (s), 767 (m), 661 (s), 599 (s). μeff (Evans method, C_6D_6, 298 K): 2.93 μ_B.

NMR Spectra

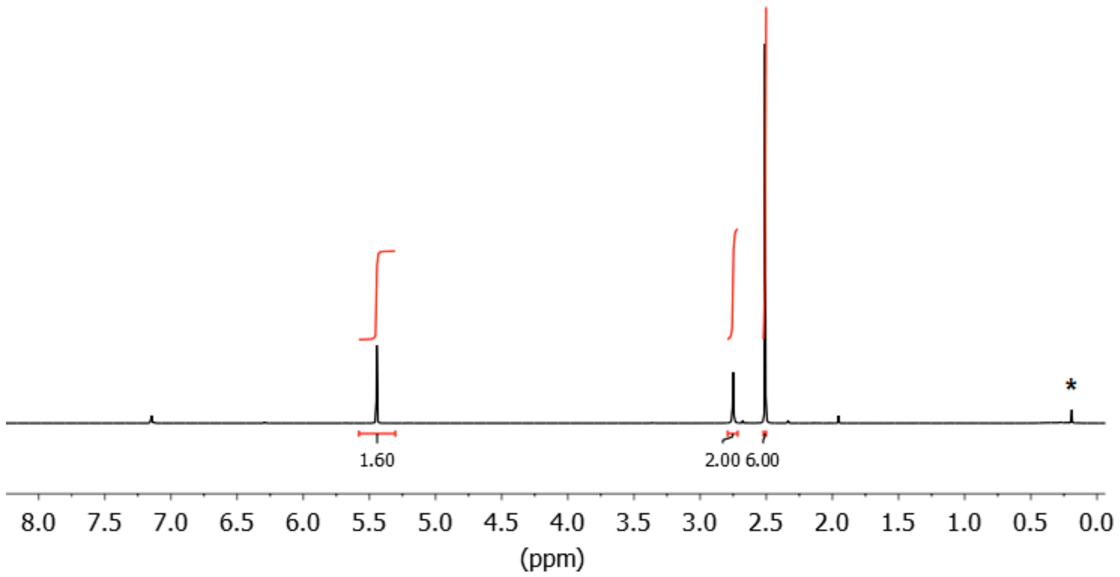

***Figure S1.** *^{1}*H NMR spectrum of 2. * = grease impurity.*

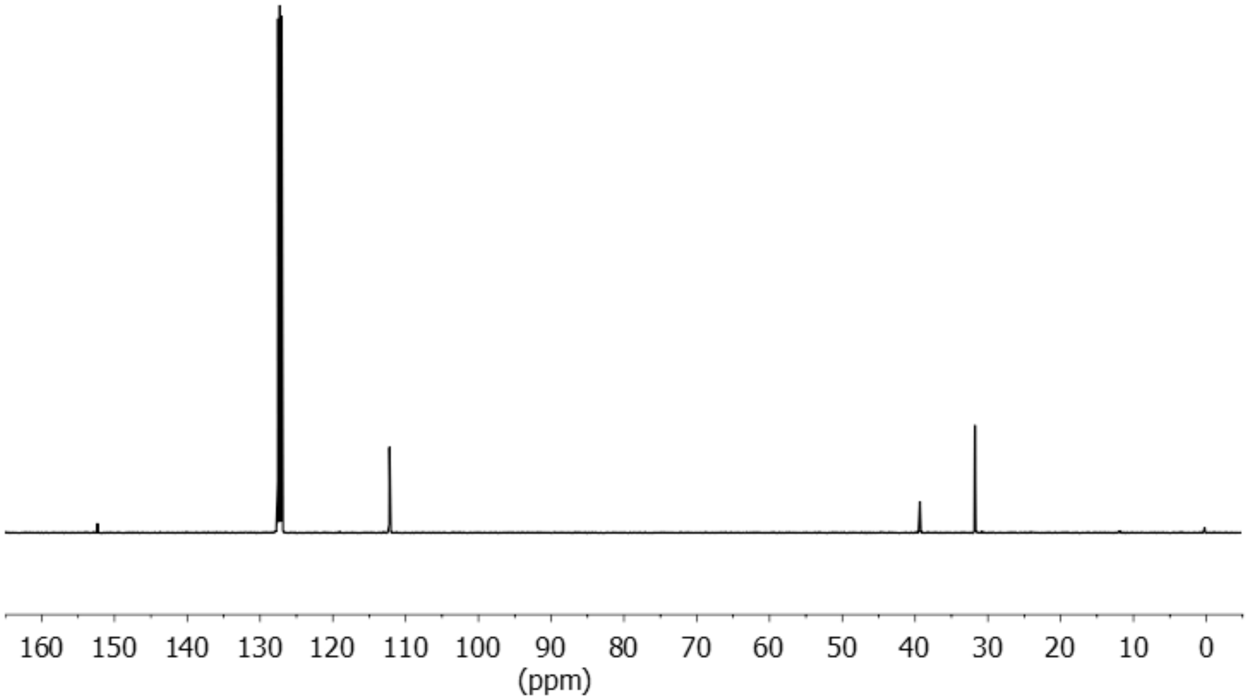

Figure S2. ***$^{13}C\{^{1}H\}$ NMR spectrum of 2.***

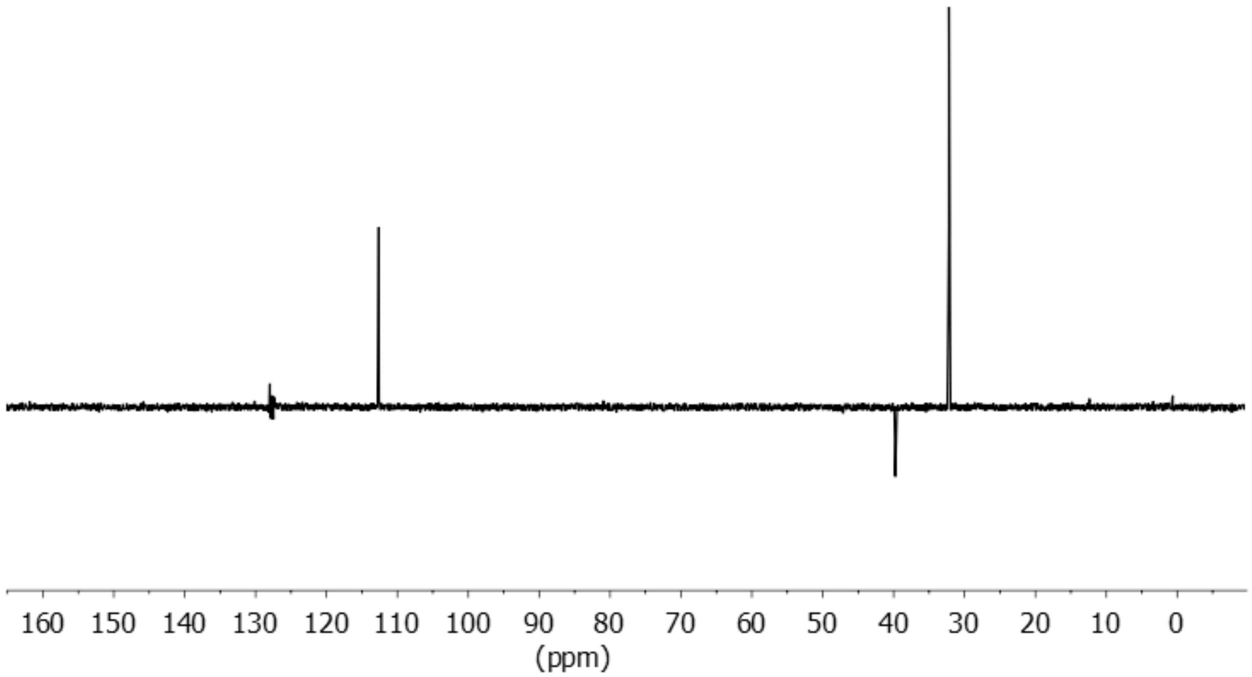

Figure S3. DEPT-135 ^{13}C NMR spectrum of 2.

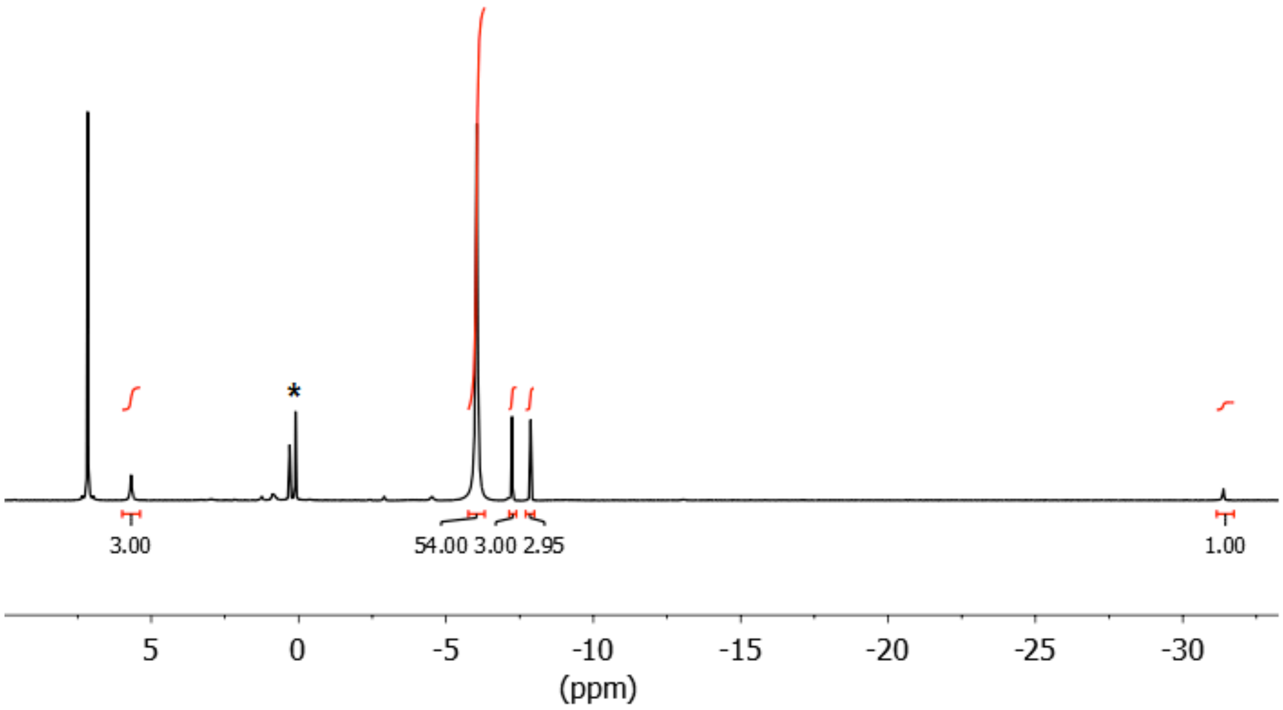

Figure S4. ***^{1}H NMR spectrum of 3U. * = grease impurity. The resonances at 7.26 and 0.3 ppm are benzene solvent and trace HN(SiMe$_3$)$_2$, the latter often observed when pure derivatives of reactive 1U are dissolved.***

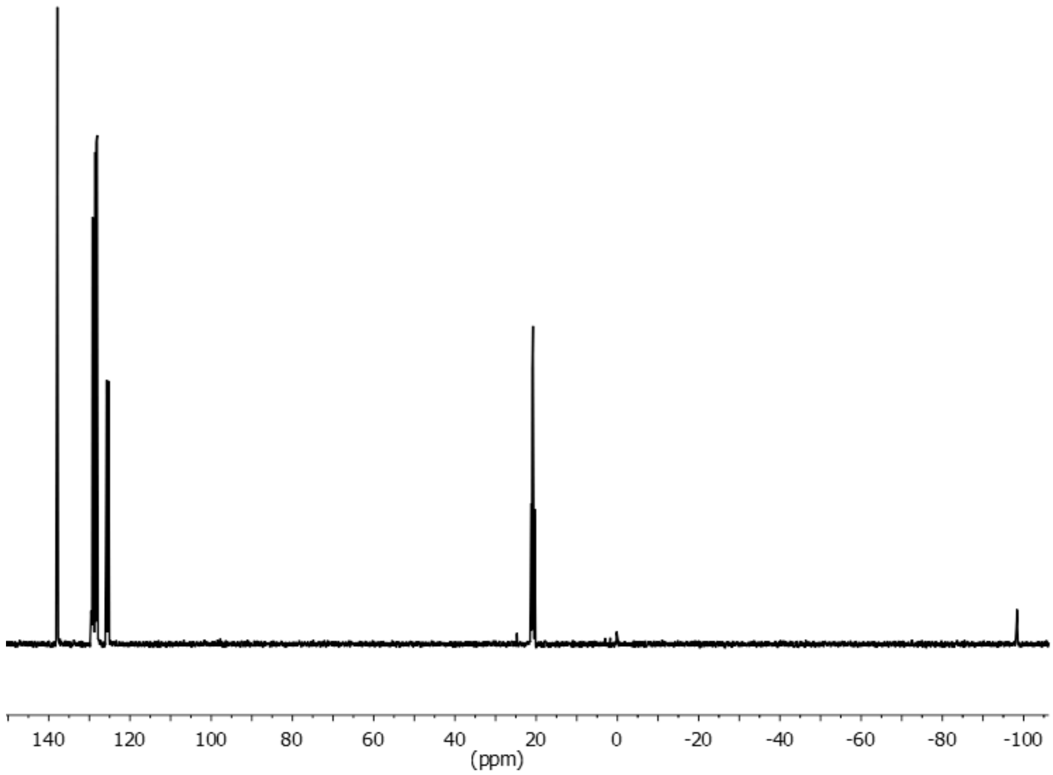

Figure S5. ***^{13}C{^{1}H} NMR spectrum of 3U.***

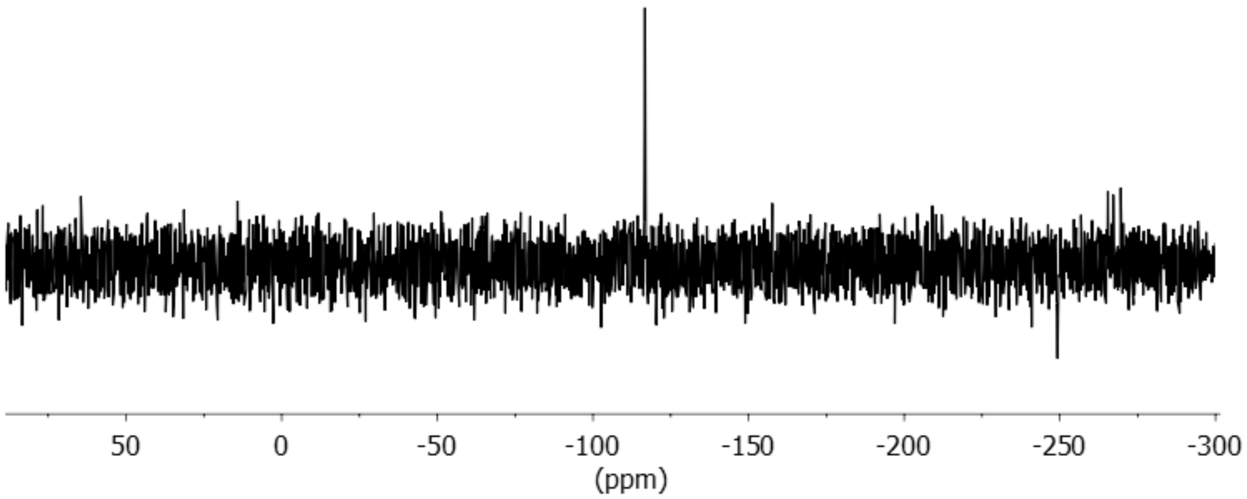

Figure S6. ***^{29}Si{^{1}H} NMR spectrum of 3U.***

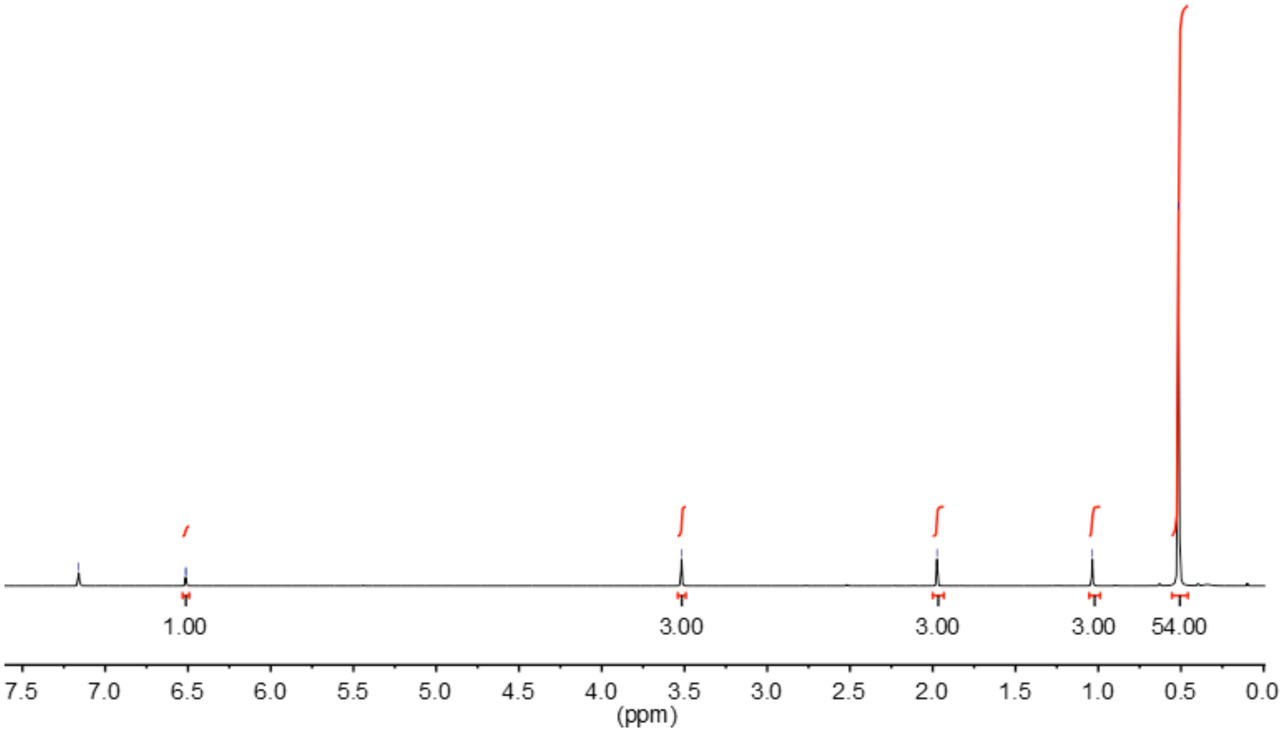

Figure S7. ***^{1}H NMR spectrum of 3Y.***

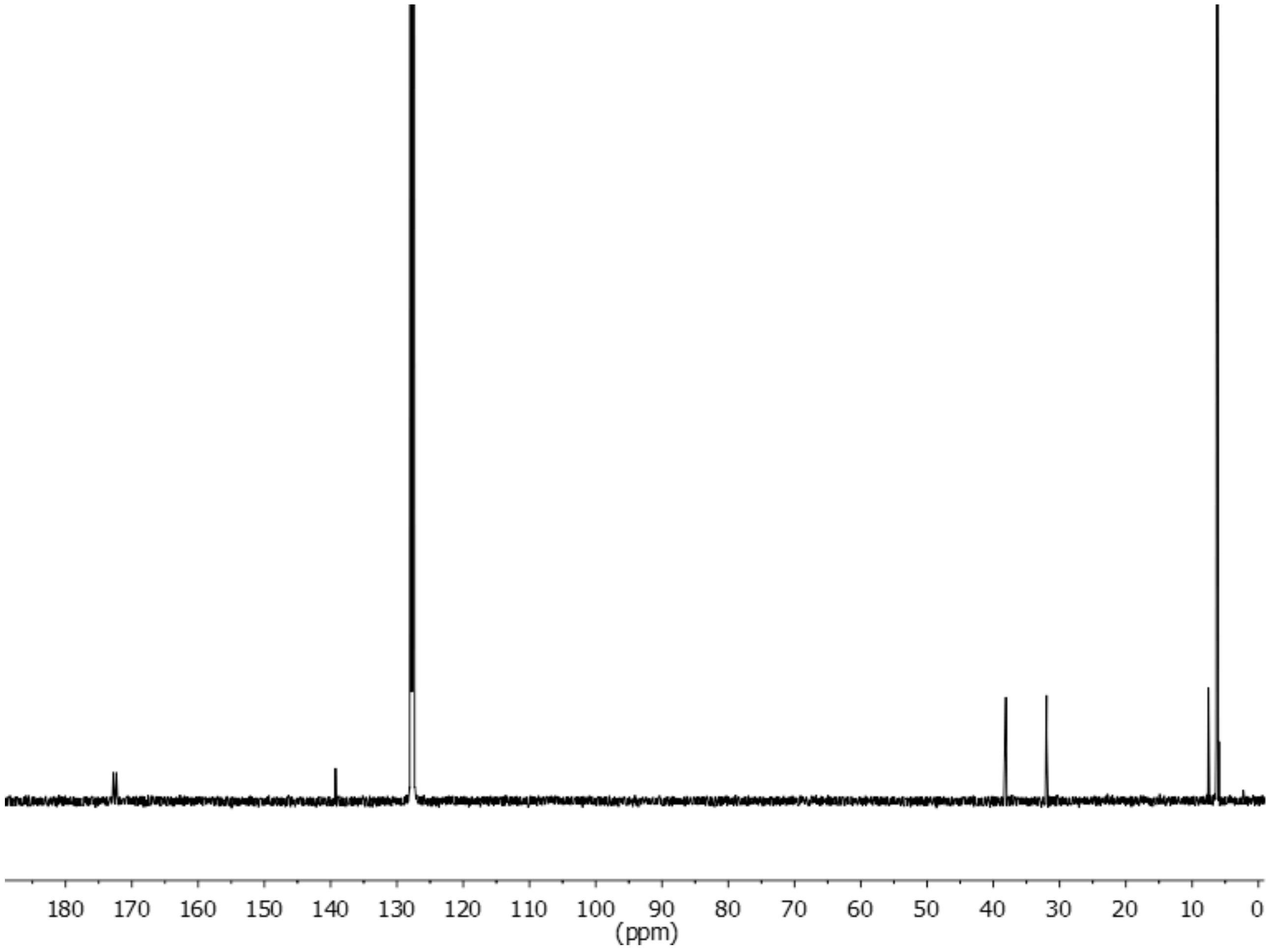

Figure S8. ***$^{13}C\{^1H\}$ NMR spectrum of 3Y.***

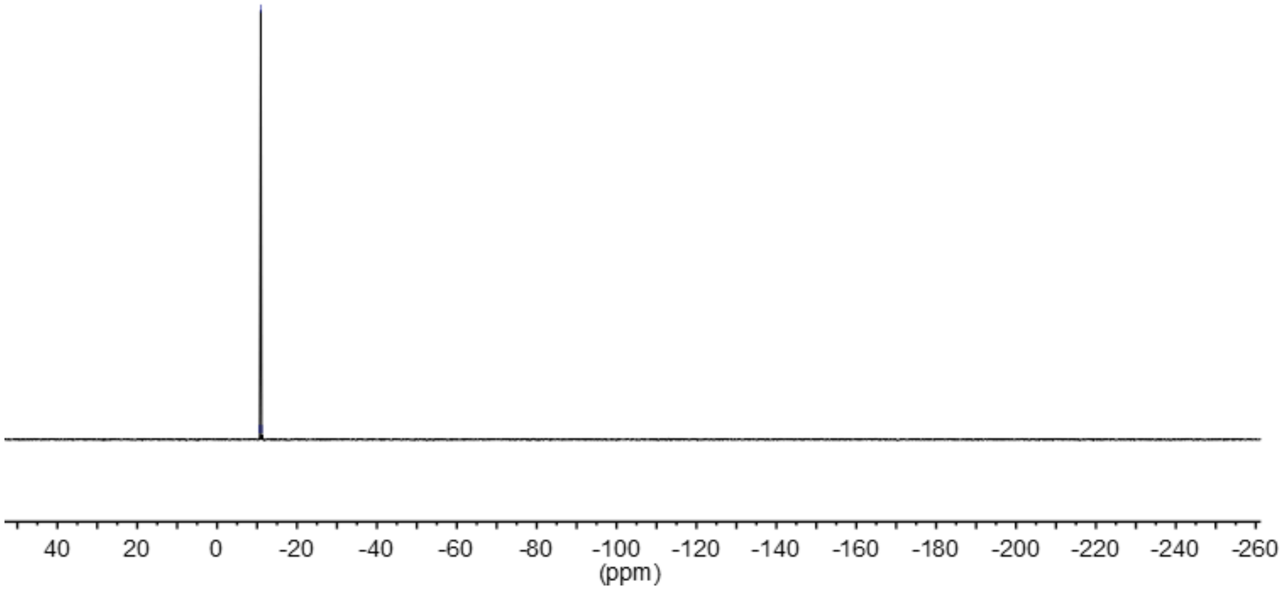

Figure S9. ***$^{29}Si\{^1H\}$ NMR spectrum of 3Y.***

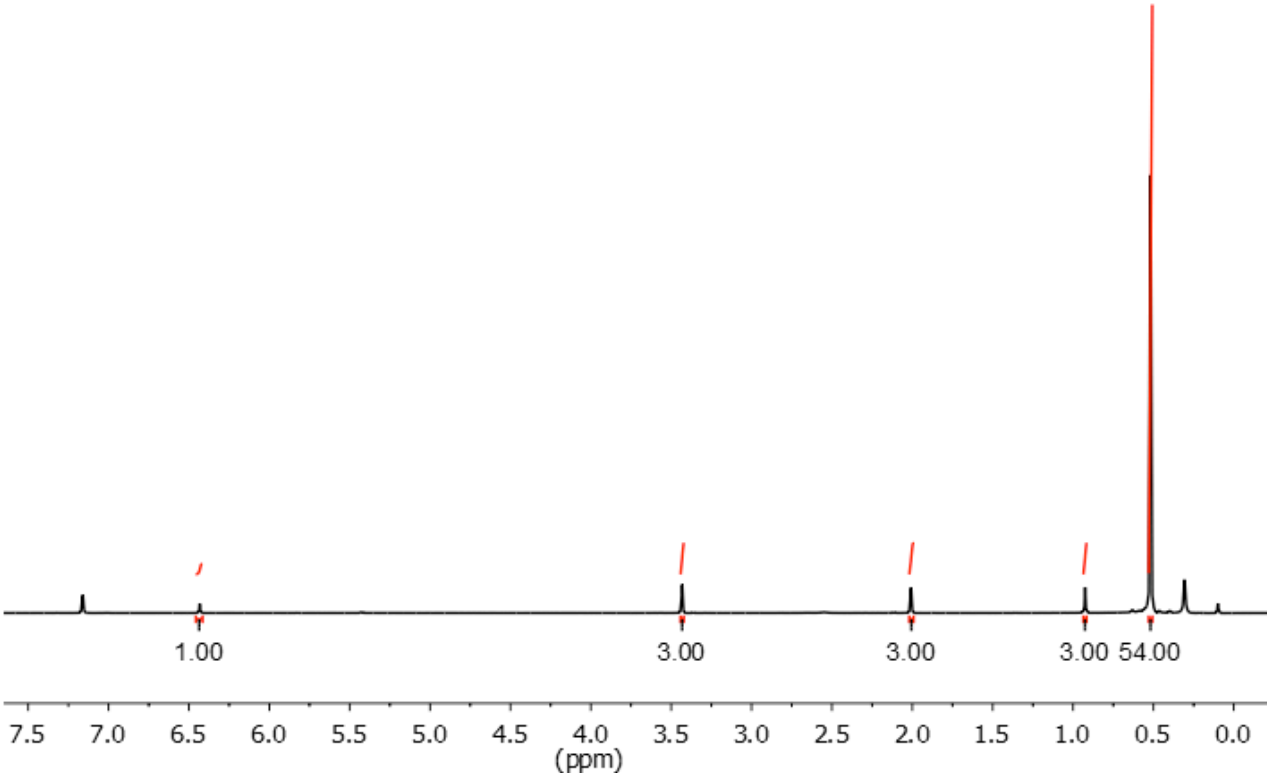

Figure S10. ***^{1}H NMR spectrum of 3La.***

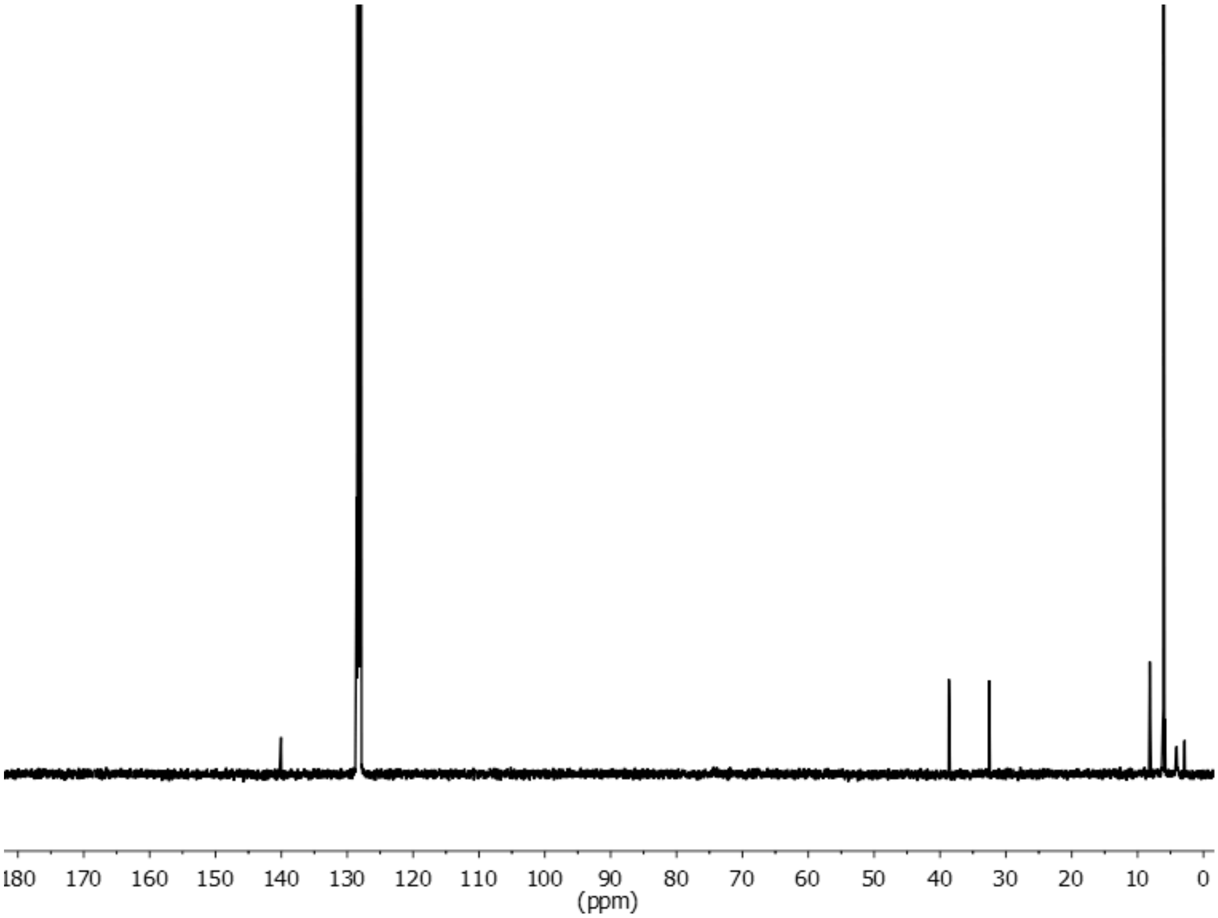

Figure S11. ***^{13}C{^{1}H} NMR spectrum of 3La.***

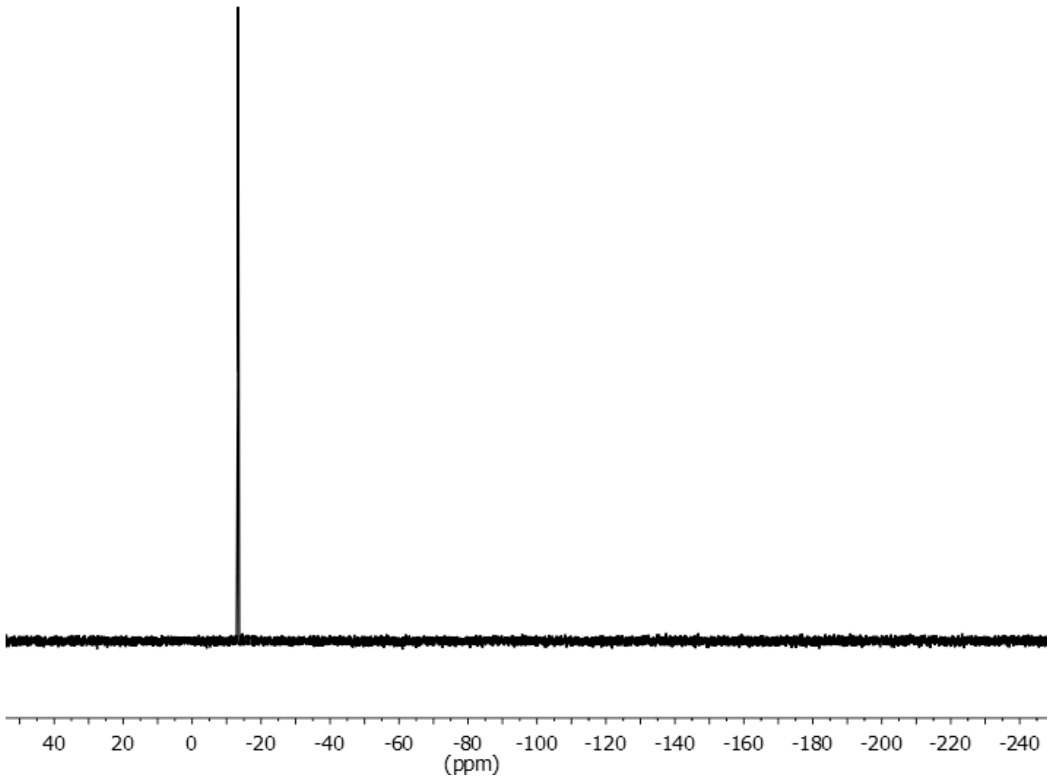

Figure S12. $^{29}Si\{^{1}H\}$ NMR spectrum of 3La.

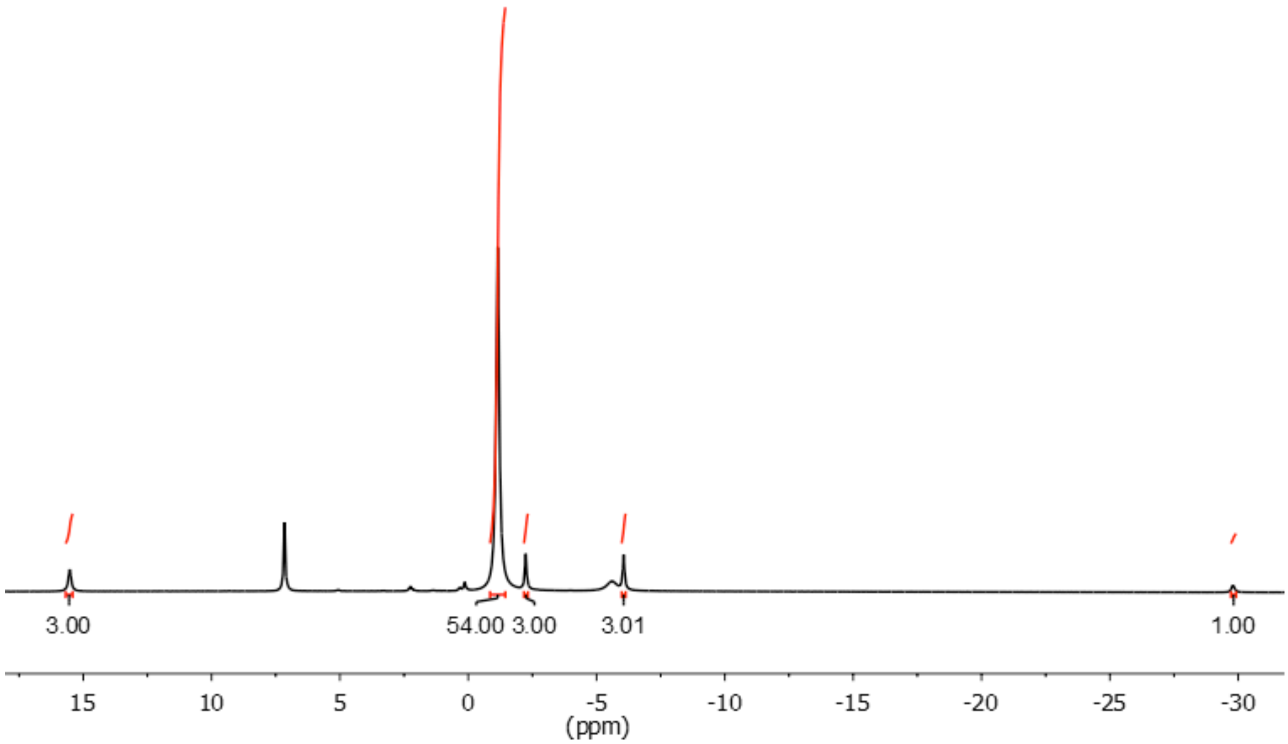

Figure S13. ^{1}H NMR spectrum of 3Nd.

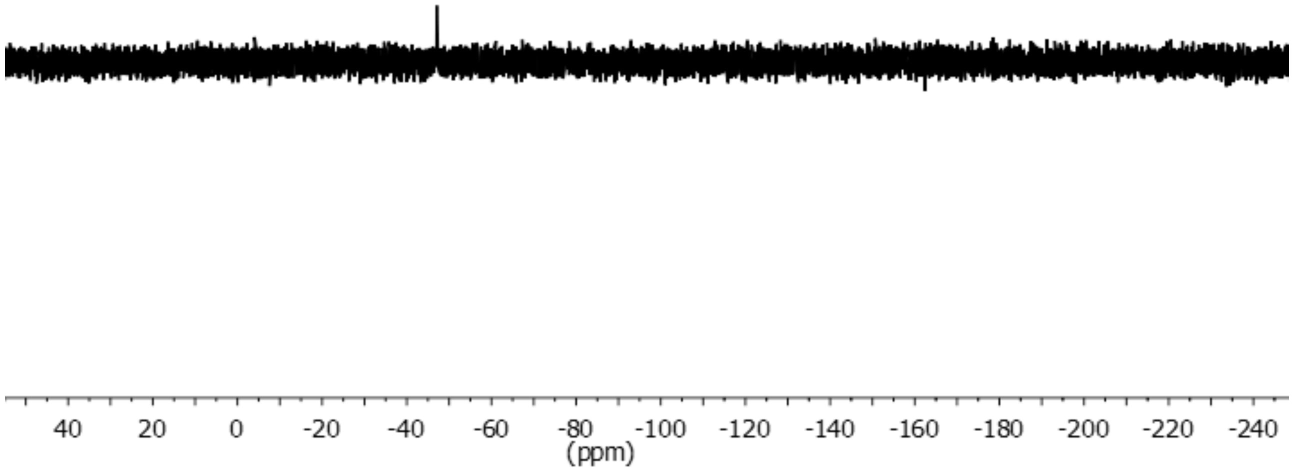

Figure S14. $^{29}Si\{^{1}H\}$ NMR spectrum of 3Nd.

ATR-IR Spectra

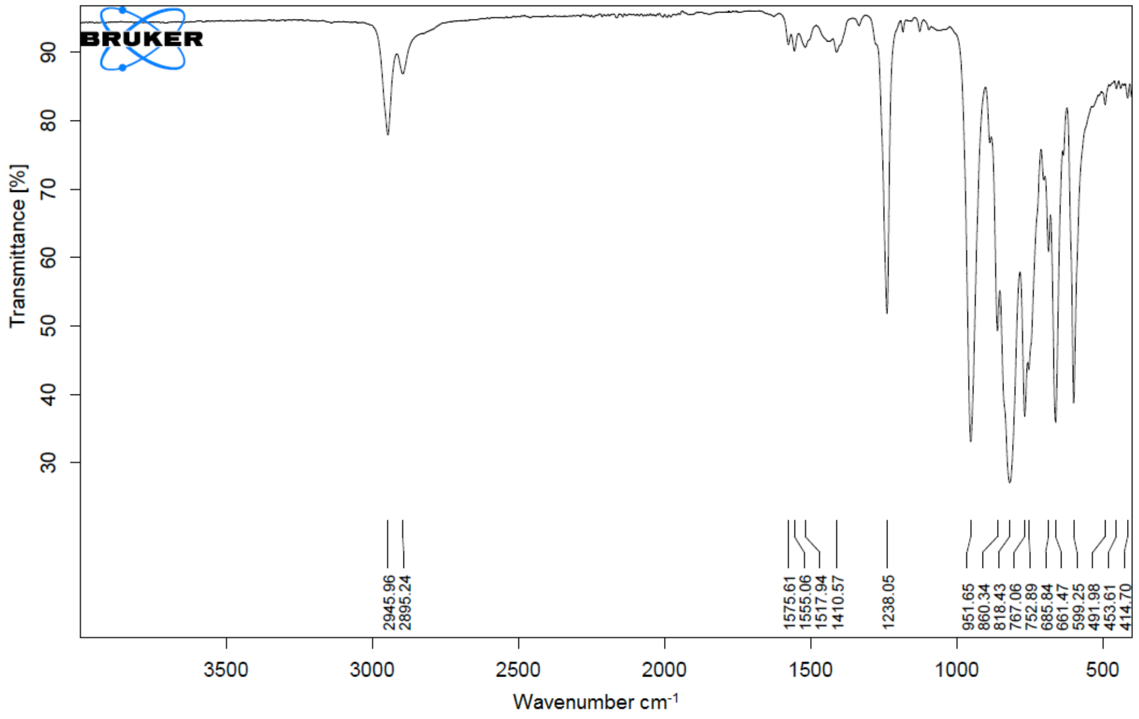

Figure S15. ATR-IR spectrum of 3U.

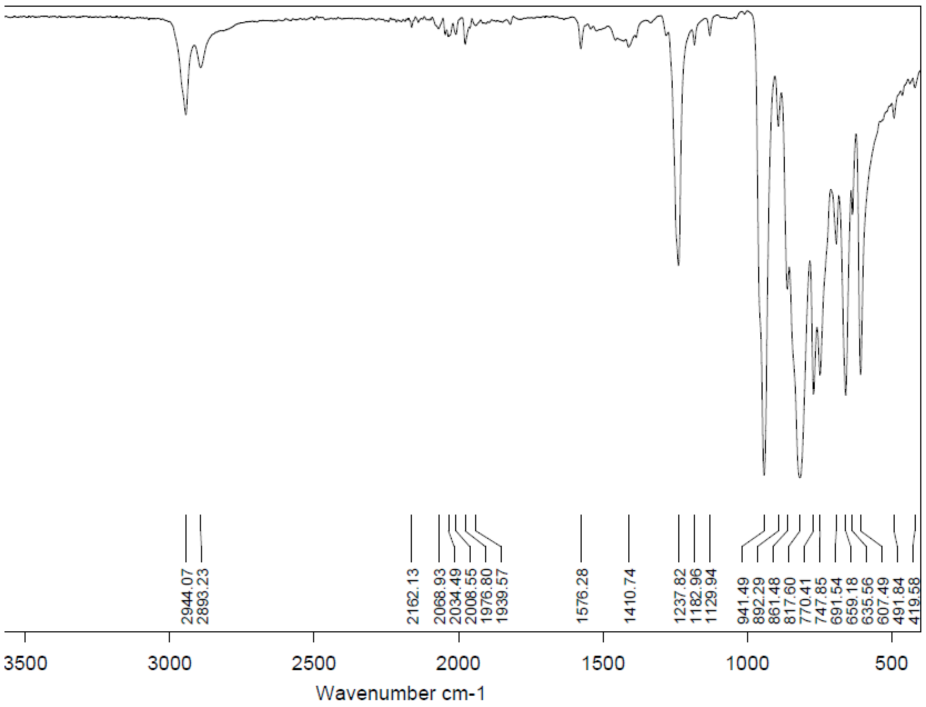

Figure S16. ATR-IR spectrum of 3Y.

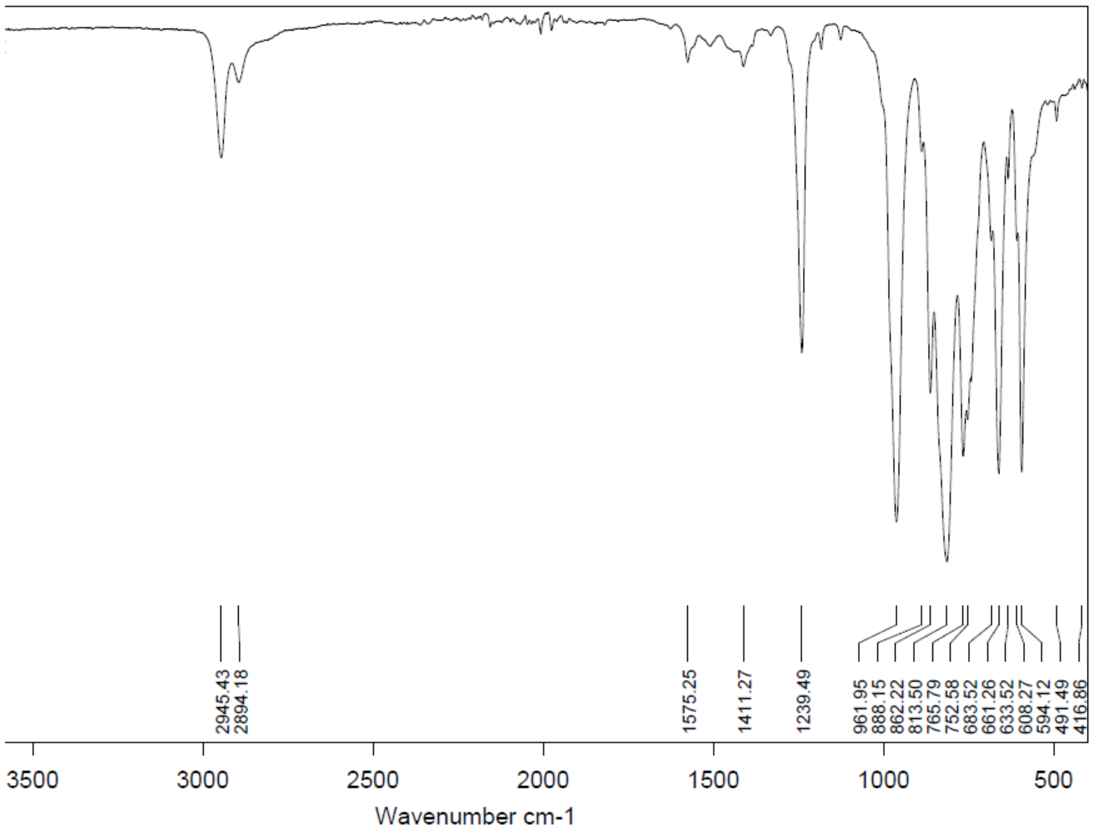

Figure S17. ATR-IR spectrum of 3La.

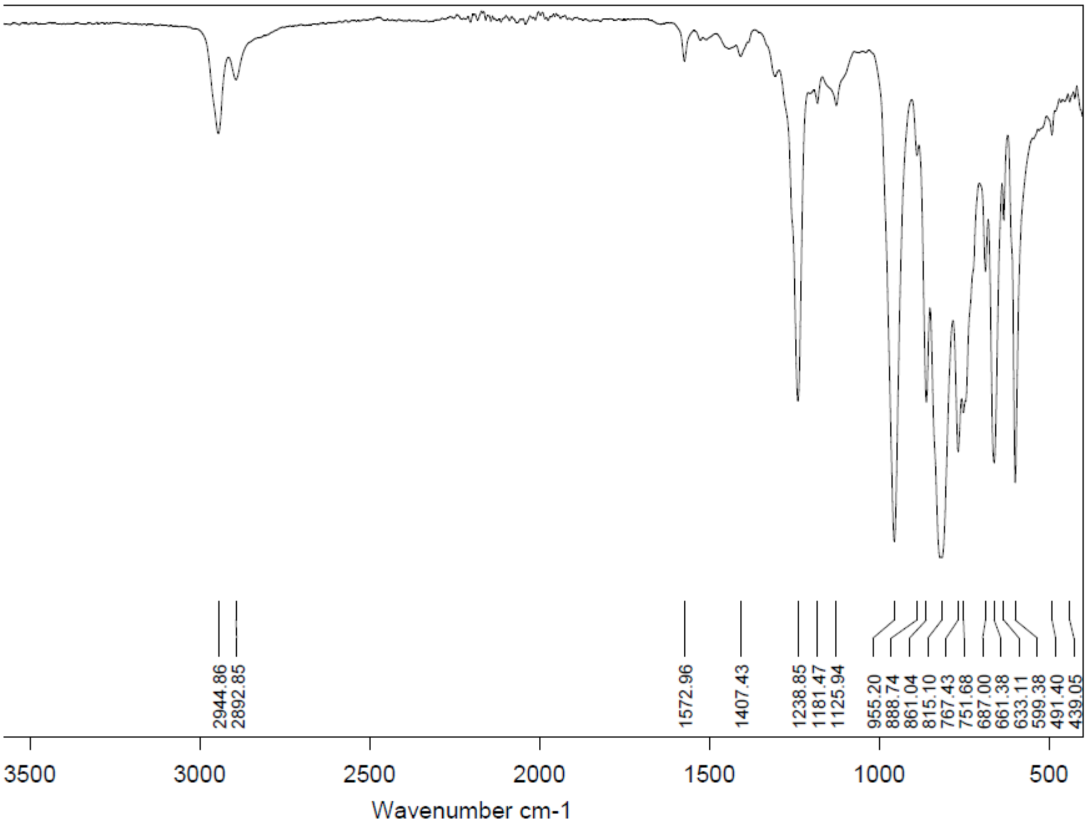

Figure S18. ATR-IR spectrum of 3Nd.

SQUID Magnetometry

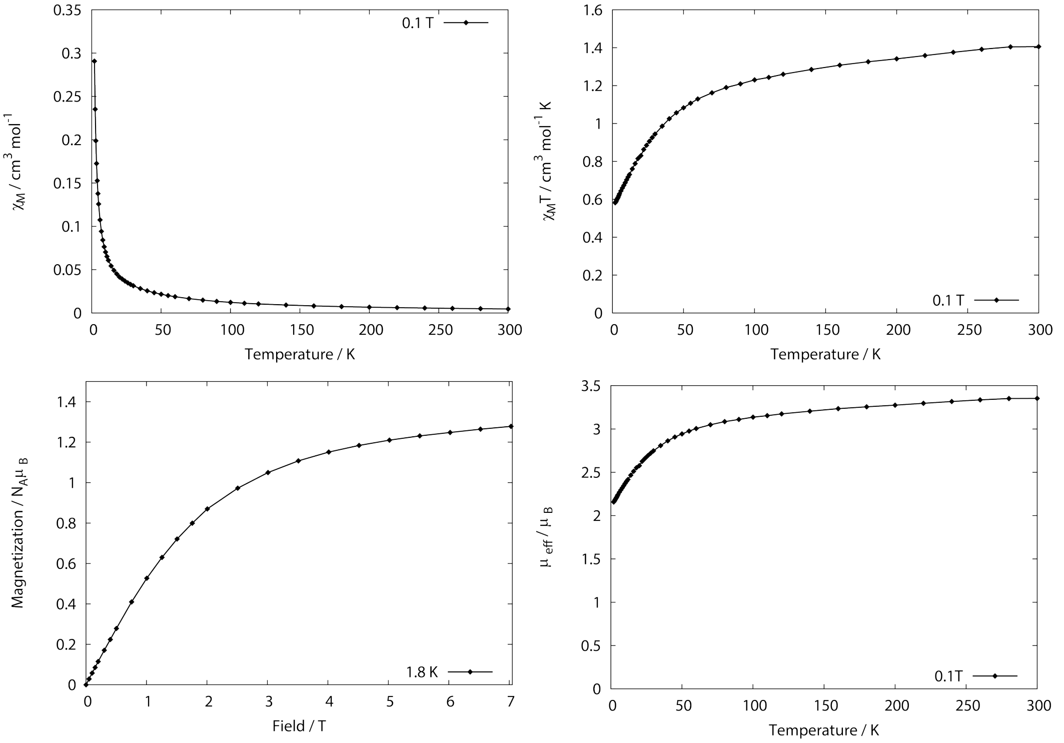

Figure S19. χ vs T, χT vs T, M vs H and μ_{eff} vs T plots for 1U. Lines are a guide to the eye only.

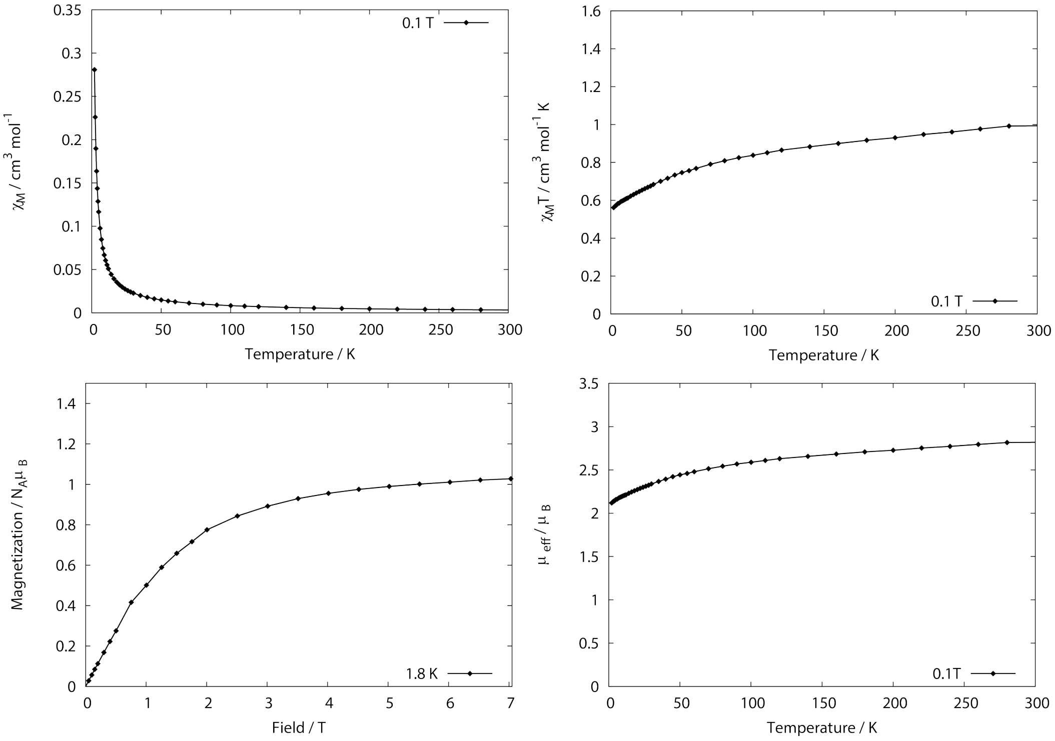

Figure S20. χ vs T, χT vs T, M vs H and μ_{eff} vs T plots for 3U. Lines are a guide to the eye only.

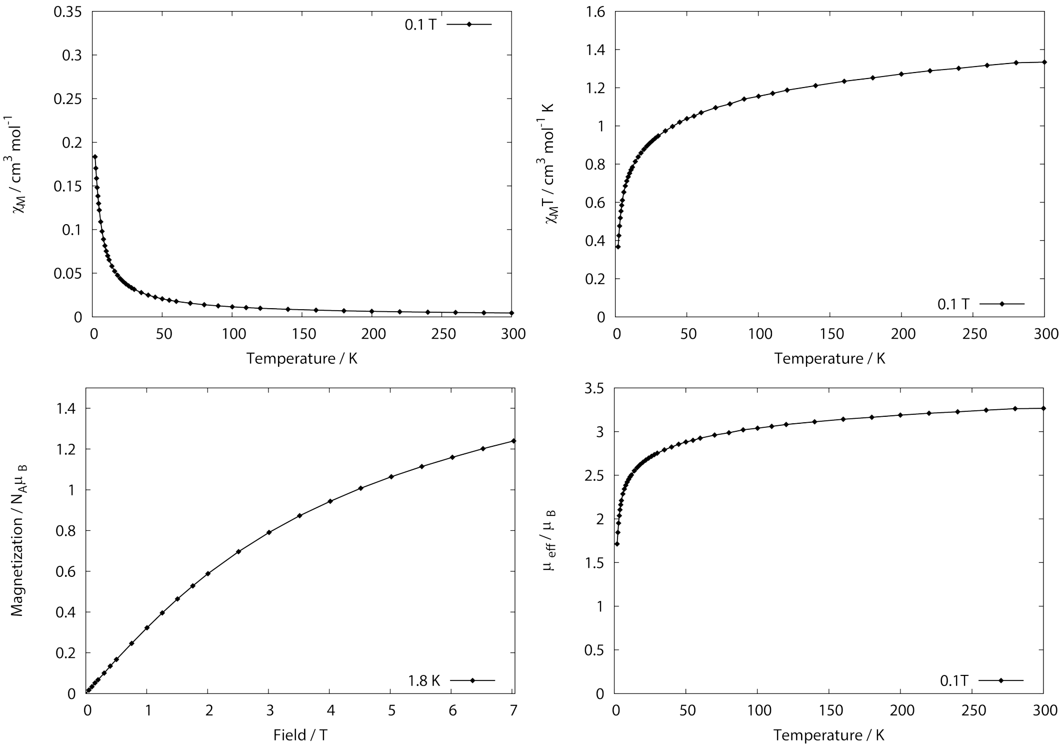

Figure S21. χ vs T, χT vs T, M vs H and μ_{eff} vs T plots for [U(N'')$_3$(I)]. Lines are a guide to the eye only.

Electronic Absorption Spectra

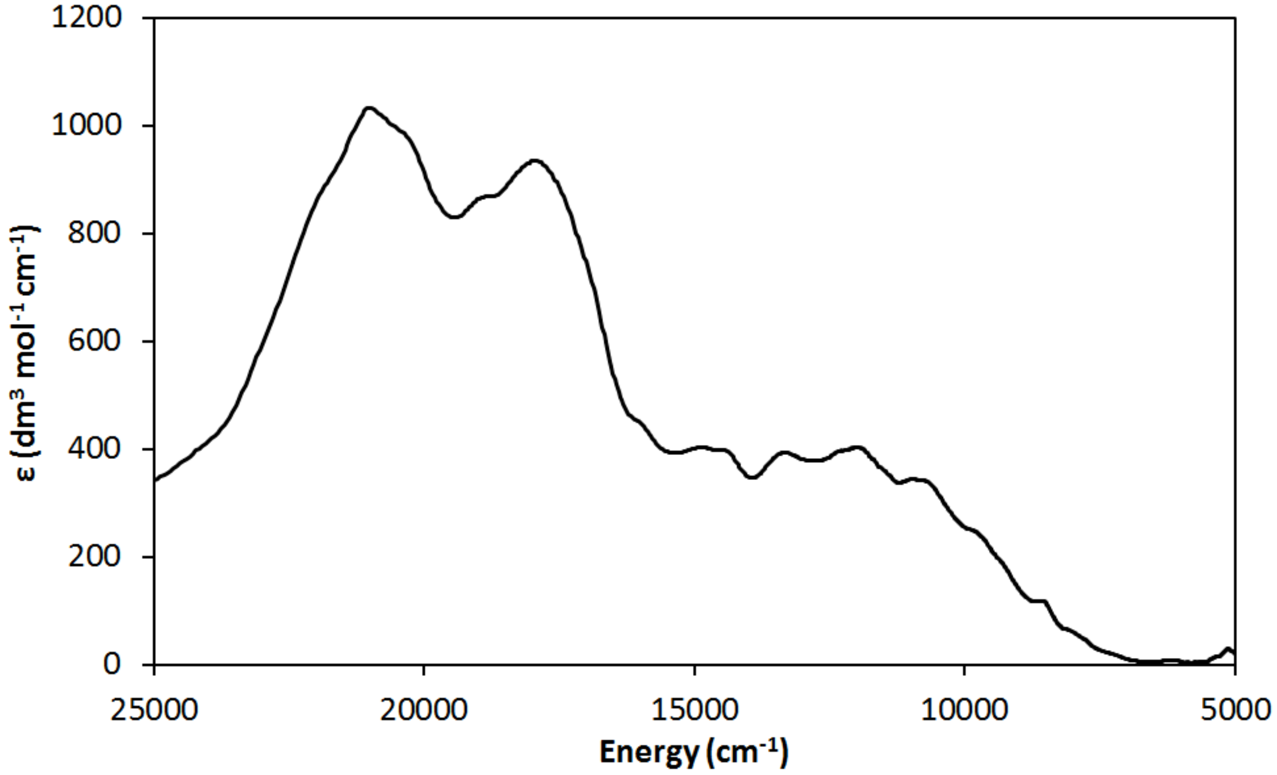

Figure S22. UV/Vis/NIR spectrum of 1U at 298 K in hexane.

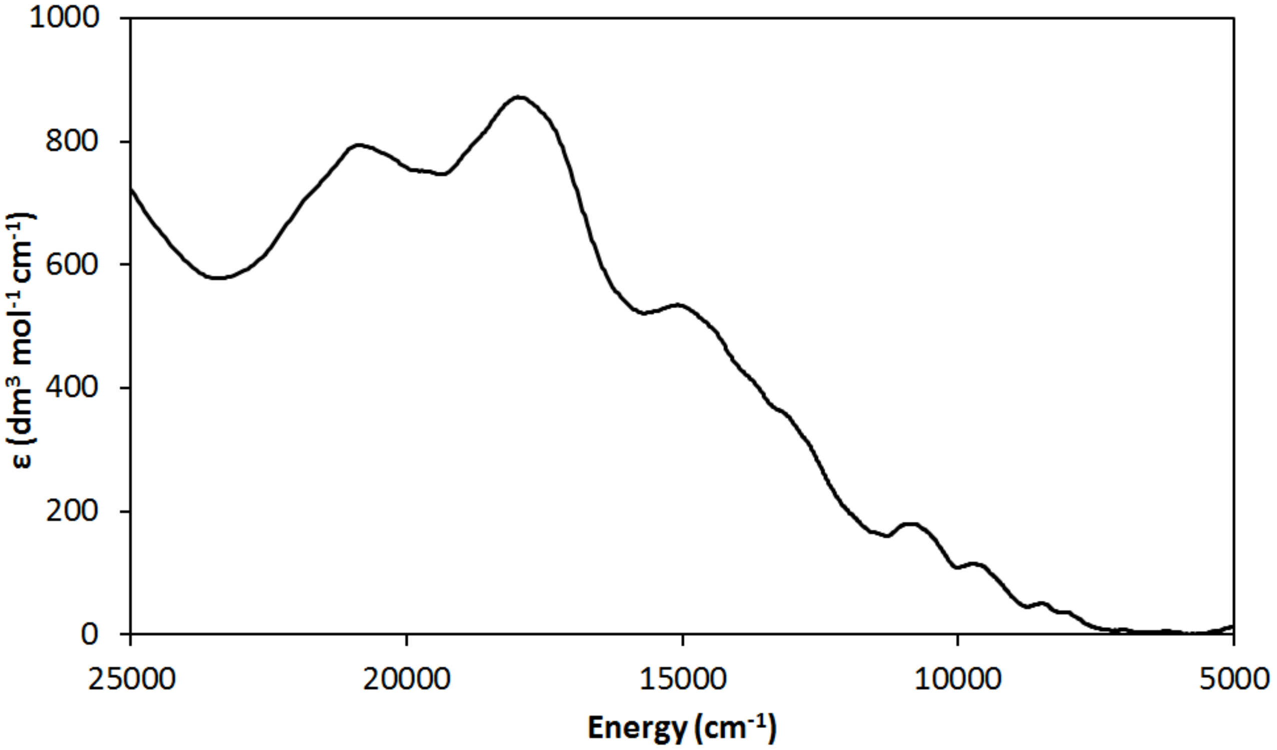

Figure S23. UV/Vis/NIR spectrum of 3U at 298 K in hexane.

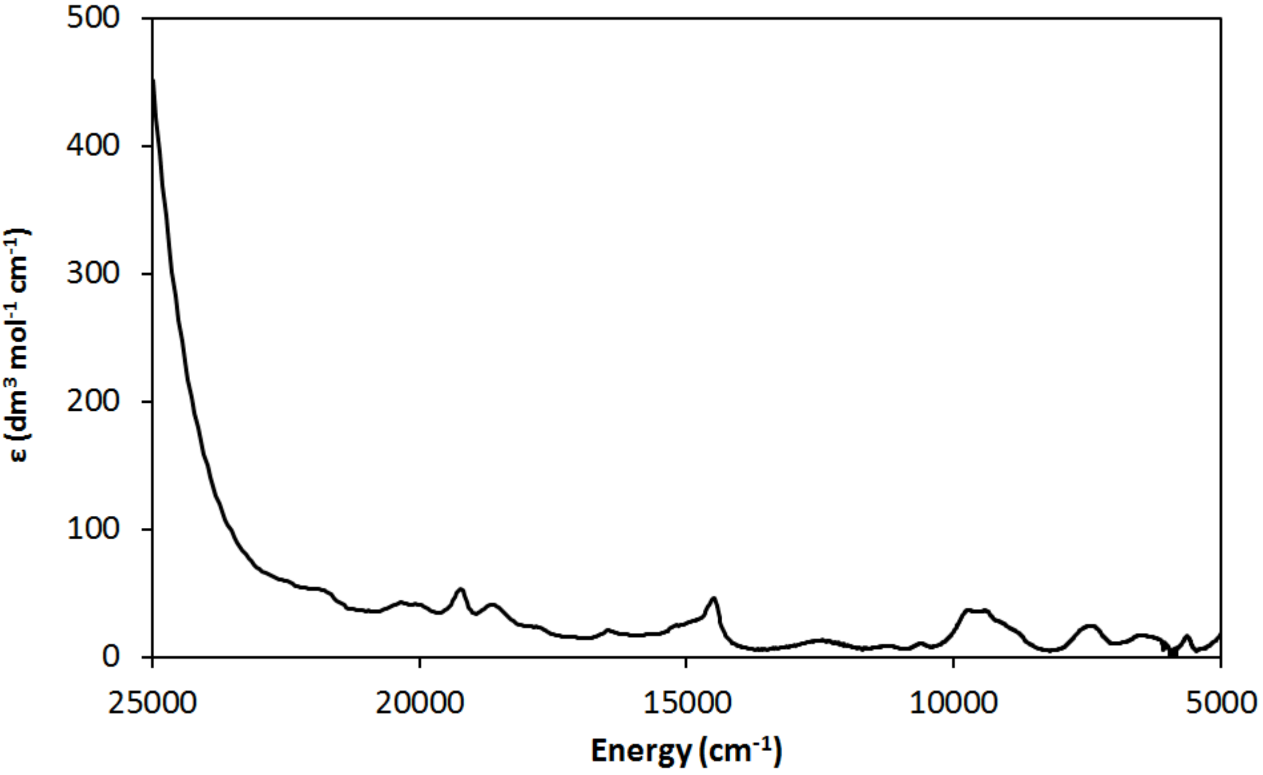

Figure S24. UV/Vis/NIR spectrum of [U(N'')$_3$(I)] at 298 K in hexane.

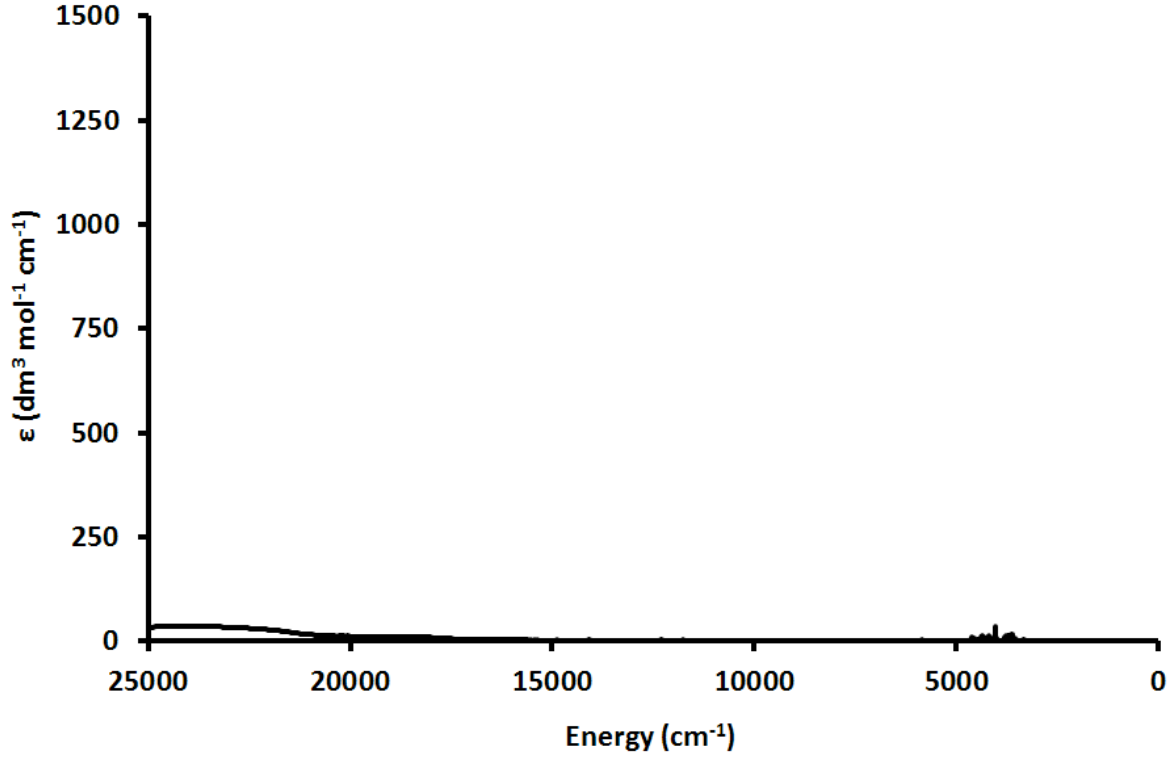

Figure S25. UV/Vis/NIR spectrum of 3Y at 298 K in hexane.

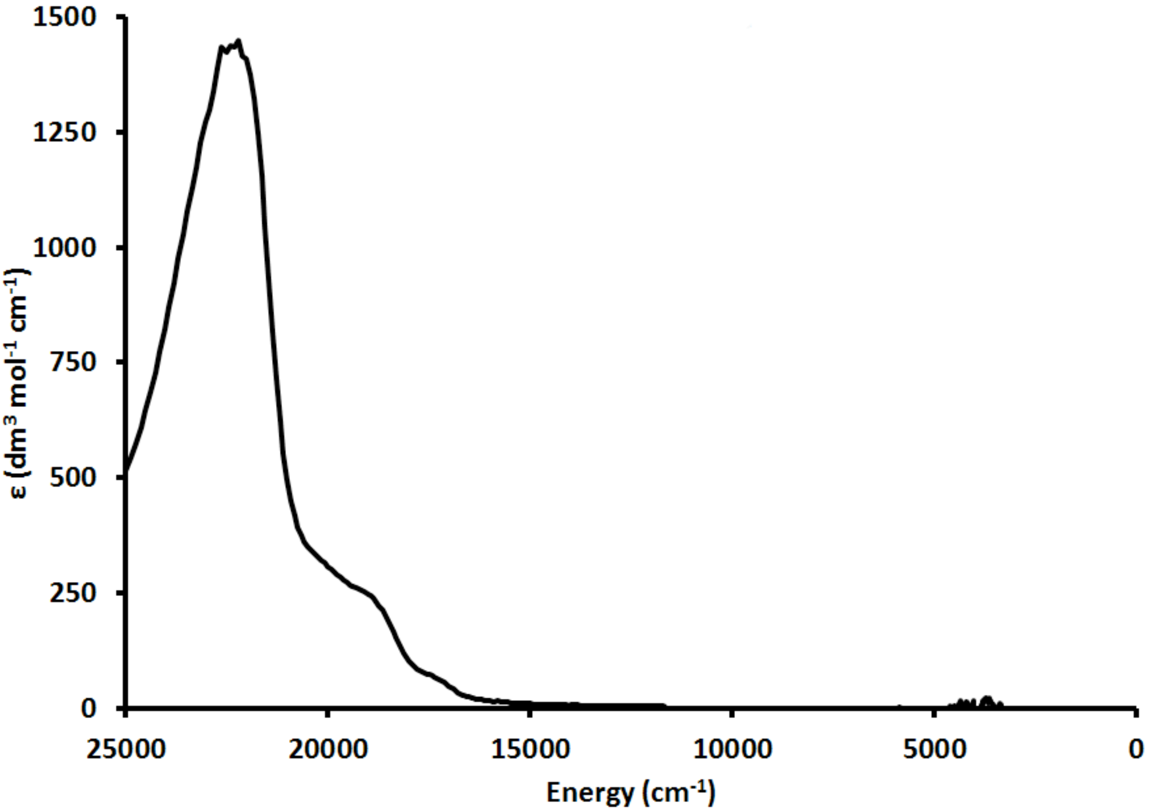

Figure S26. UV/Vis/NIR spectrum of 3La at 298 K in hexane.

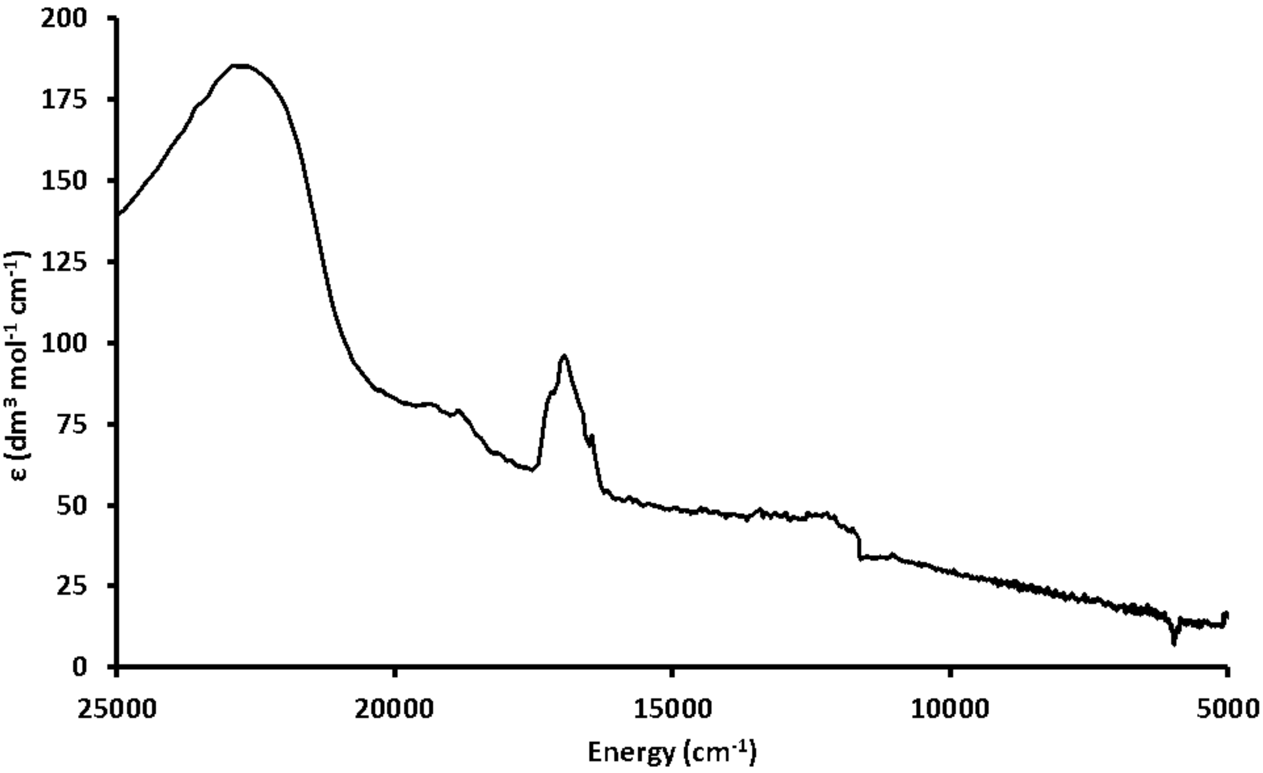

Figure S27. UV/Vis/NIR spectrum of 3Nd at 298 K in hexane.

X-ray Crystallography

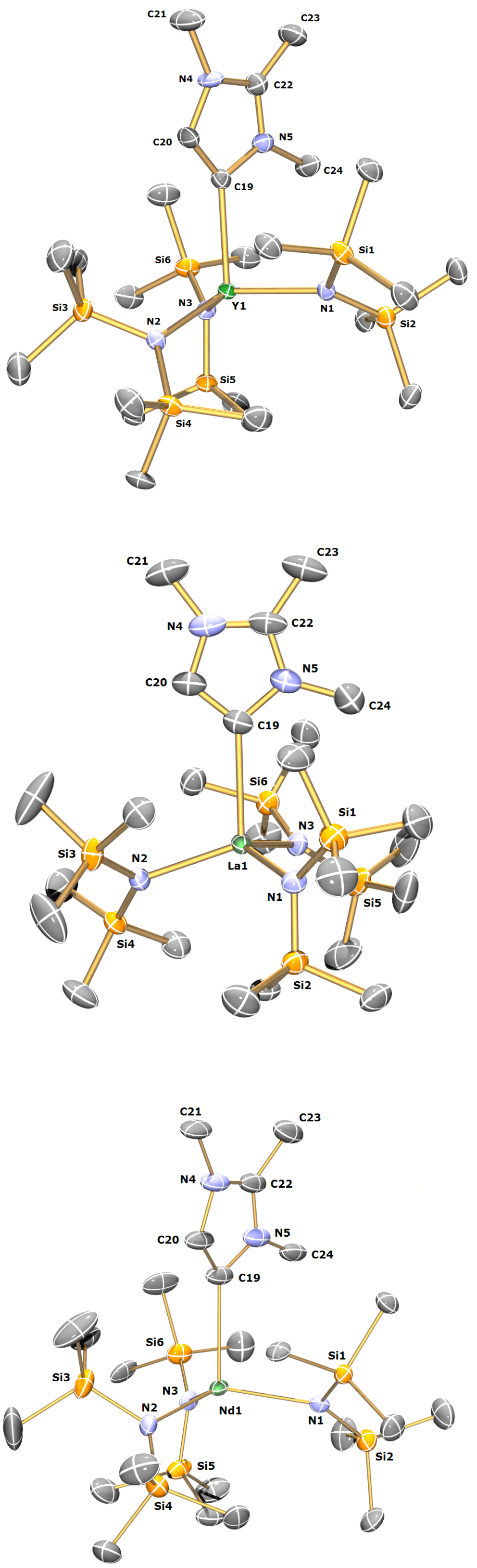

***Figure S28.** Molecular structures of 3Y (top), 3La (middle), and 3Nd (bottom) at 150 K and displacement ellipsoids set to 40%. H-atoms and disorder components are omitted for clarity.*

Table S1. Experimental crystallographic details for 3U, 3Y, 3La, and 3Nd. CCDC numbers 1505533, 1556614, 1556615, and 1556616.

	**3U**	**3Y**	**3La**	**3Nd**
Formula	$C_{24}H_{64}N_5Si_6U$	$C_{24}H_{64}N_5Si_6Y$	$C_{24}H_{64}LaN_5Si_6$	$C_{24}H_{64}N_5NdSi_6$
Fw	829.37	680.25	730.25	735.58
Cryst size, mm^3	0.81 x 0.41 x 0.29	0.40 x 0.20 x 0.13	0.45 x 0.22 x 0.16	0.44 x 0.32 x 0.20
Cryst syst	Monoclinic	Orthorhombic	Monoclinic	Triclinic
Space group	$P2_1/c$	$Pna2_1$	$P2_1/c$	P-1
Collection Temperature	150(2)	125(2)	125(2)	150(2)
a, Å	11.8011(9)	23.3686(8)	11.7608(3)	11.7720(3)
b, Å	18.0886(15)	12.7341(5)	18.2196(4)	18.1324(5)
c, Å	18.9345(19)	13.1389(6)	18.6831(4)	18.6344(5)
α, °				90.617(2)
β, °	90.654(8)		92.090(2)	91.689(2)
γ, °				91.121(2)
V, $Å^3$	4041.6(6)	3909.9(3)	4000.70(16)	3974.85(18)
Z	4	4	4	4
ρ_{calc} g cm^{-3}	1.363	1.156	1.212	1.229
μ, mm^{-1}	4.214	1.696	1.267	1.507
no. of reflections measd	28064	15016	17838	49263
no. of unique reflns, Rint	7121, 0.0938	6120, 0.0479	9069, 0.0324	14010, 0.0533
no. of reflns with $F^2 > 2s(F^2)$	5188	5042	7351	12558
transmn coeff range	0.14-0.41	0.64-0.83	0.73-0.87	0.36-1.00
R, R_w^a ($F^2 > 2s(F^2)$)	0.0582, 0.1230	0.0408, 0.0783	0.0463, 0.0851	0.1034, 0.2490
R, R_w^a (all data)	0.0874, 0.1395	0.0608, 0.0860	0.0624, 0.0914	0.1108, 0.2530
S^a	1.044	1.033	1.060	1.170
Parameters	497	347	422	1108
max.,min. diff map, e $Å^{-3}$	1.301, -1.492	0.458, -0.382	0.707, -0.488	7.595, -1.761

Table S2. Experimental bond lengths (Å) and angles (°) for 3U, 3Y, 3La, and 3Nd.

**3U**			
U1-N1	2.379(7)	U1-N2	2.359(7)
U1-N3	2.381(7)	U1-C19	2.598(11)
U1-C19A	2.590(13)	U1-C19B	2.576(12)
C19-C20	1.373(9)	C19A-C20A	1.371(10)
C19B-C20B	1.362(10)	N5-C19	1.429(9)
N5A-C19A	1.425(9)	N5B-C19B	1.422(9)
N4-C20	1.375(9)	N4A-C20A	1.378(9)
N4B-C20B	1.383(9)	N4-C22	1.335(9)
N4A-C22A	1.335(9)	N4B-C22B	1.331(9)
N5-C22	1.337(9)	N5A-C22A	1.339(9)
N5B-C22B	1.351(9)	C22-C23	1.492(9)
C22A-C23A	1.490(9)	C22B-C23B	1.488(9)
N4-C21	1.511(9)	N4A-C21A	1.498(9)
N4B-C21B	1.498(9)	N5-C24	1.500(9)
N5A-C24A	1.495(9)	N5B-C24B	1.485(9)
N1-U1-C19	110.6(4)	N1-U1-C19A	114.1(5)
N1-U1-C19B	106.4(4)	N2-U1-C19	106.6(4)
N2-U1-C19A	106.5(11)	N2-U1-C19B	115.6(5)
N3-U1-C19	91.3(4)	N3-U1-C19A	87.7(9)
N3-U1-C19B	86.4(4)	N1-U1-N2	109.8(2)
N2-U1-N3	117.5(2)	N1-U1-N3	118.7(2)
**3Y**			
Y1-N1	2.266(4)	Y1-N2	2.278(5)
Y1-N3	2.268(5)	Y1-C19	2.495(7)
C19-C20	1.349(8)	N5-C19	1.412(8)
N4-C20	1.385(8)	N4-C22	1.330(8)
N5-C22	1.349(8)	C22-C23	1.472(9)
N4-C21	1.473(8)	N5-C24	1.467(7)
N1-Y1-C19	92.18(19)	N2-Y1-C19	114.14(19)
N3-Y1-C19	102.59(18)	N1-Y1-N2	118.71(16)
N2-Y1-N3	108.56(17)	N1-Y1-N3	118.48(17)
**3La**			
La1-N1	2.418(3)	La1-N2	2.415(3)
La1-N3	2.413(3)	La1-C19	2.699(5)
La1-C19A	2.675(14)	C19A-C20A	1.357(10)
C19-C20	1.381(6)	N5-C19	1.410(5)
N5A-C19A	1.400(9)	N4-C20	1.365(5)
N4A-C22A	1.324(9)	N4A-C20A	1.386(10)
N5-C22	1.330(6)	N4-C22	1.341(6)
C22A-C23A	1.468(9)	N5A-C22A	1.354(8)
N4-C21	1.467(5)	C22-C23	1.478(6)
N5A-C24A	1.463(9)	N4A-C21A	1.474(10)
N5-C24	1.462(5)	N1-La1-C19	93.54(16)
N2-La1-C19A	101.6(7)	N1-La1-C19A	86.7(6)
N3-La1-C19	107.55(13)	N2-La1-C19	107.68(16)

N2-La1-N3	112.24(10)	N3-La1-C19A	119.9(4)
N1-La1-N3	117.40(10)	N1-La1-N2	115.91(10)
**3Nd**			
Nd1-N1	2.386(8)	Nd1-N2	2.388(9)
Nd1-N3	2.399(8)	Nd1-C19	2.615(12)
Nd1A-C19A	2.625(12)	Nd1A-N1A	2.444(8)
Nd1A-N2A	2.423(8)	Nd1A-N3A	2.391(8)
Nd2-N7	2.381(6)	Nd2-N6	2.374(6)
Nd2-N8	2.375(6)	Nd2-C43	2.620(11)
Nd2-C43A	2.614(12)	C19A-C20A	1.42(2)
C19-C20	1.40(2)	N5A-C19A	1.366(18)
N5-C19	1.36(2)	N4A-C20A	1.361(17)
N4-C20	1.34(2)	N5A-C22A	1.323(18)
N5-C22	1.32(2)	N4A-C21A	1.448(18)
N4-C21	1.45(2)	N5A-C24A	1.41(2)
N5-C24	1.42(2)	C22A-C23A	1.467(19)
C22-C23	1.47(2)	N4A-C22A	1.34(2)
N4-C22	1.36(2)	C43A-C44A	1.41(2)
C43-C44	1.41(2)	N10A-C43A	1.367(19)
N10-C43	1.367(18)	N9A C44A	1.364(18)
N9-C44	1.364(18)	N10A-C46A	1.323(19)
N10-C46	1.321(19)	C46A-C47A	1.47(2)
C46-C47	1.47(2)	N9A-C45A	1.448(19)
N9-C45	1.447(19)	N9A-C46A	1.34(2)
N9-C46	1.34(2)	N10A-C48A	1.41(2)
N10-C48	1.41(2)	N2-Nd1-C19	107.7(9)
N1-Nd1-C19	103.8(7)	N1-Nd1-N3	95.0(7)
N3-Nd1-C19	88.2(9)	N2-Nd1-N3	139.2(6)
N1-Nd1-N2	116.0(7)	N2A-Nd1A-C19A	90.9(7)
N1A-Nd1A-C19A	104.6(7)	N1A-Nd1A-N3A	115.0(6)
N3A-Nd1A-C19A	109.7(7)	N2A-Nd1A-N3A	133.9(6)
N1A-Nd1A-N2A	97.6(5)	N7-Nd2-C43	92.6(5)
N6-Nd2-C43	108.0(5)	N6-Nd2-N8	112.4(3)
N8-Nd2-C43	106.3(5)	N8-Nd2-N7	117.4(3)
N6-Nd2-N7	117.1(3)	N7-Nd2-C43A	86.6(6)
N6-Nd2-C43A	100.7(6)	N8-Nd2-C43A	119.3(6)

DFT Calculations

General

Unrestricted and restricted geometry optimisations were performed as appropriate for the full models of **3U**, **3Y**, **3La**, and **3Nd** using coordinates derived from their X-ray crystal structures where one of the MIC disorder components for **3U**, **3La**, and **3Nd** was arbitrarily selected as a start-point. No constraints were imposed on the structures during the geometry optimisations. The calculations were performed using the Amsterdam Density Functional (ADF) suite version 2012.01.[5,6] The DFT geometry optimisations employed Slater type orbital (STO) triple-ζ-plus polarisation all-electron basis sets (from the ZORA/TZP database of the ADF suite). Scalar relativistic approaches were used within the ZORA Hamiltonian for the inclusion of relativistic effects and the local density approximation (LDA) with the correlation potential due to Vosko *et al* was used in all of the calculations.[7] Gradient corrections were performed using the functionals of Becke and Perdew.[8,9] MOLEKEL[10] was used to prepare the three-dimensional plots of the electron density. Natural Bond Order (NBO) analyses were carried out with NBO 5.0.[11] The Atoms in Molecules analysis[12,13] was carried out with Xaim-1.0.[14]

Selected Kohn Sham Molecular Orbitals

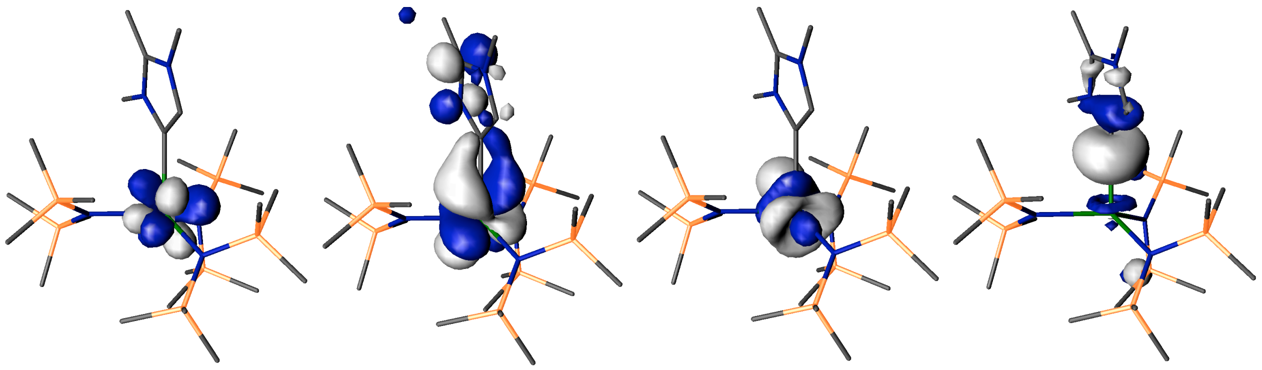

Figure S29. Left to right: HOMO (211a, −2.045 eV), HOMO−1 (210a, −2.076 eV), HOMO−2 (209a, −2.173 eV), and HOMO−16 (195a, −6.652 eV) of 3U. Hydrogen atoms are omitted for clarity.

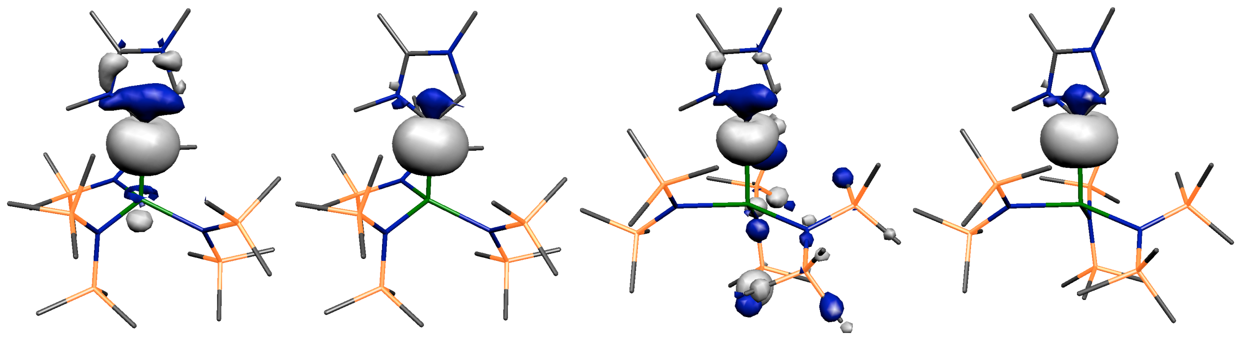

Figure S30. Left to right: HOMO−13 (170, −6.803 eV) and NBO of the Y-C σ-bond of 3Y, HOMO−10 (182, −6.415 eV) and NBO of the La-C σ-bond of 3La. Hydrogen atoms are omitted for clarity.

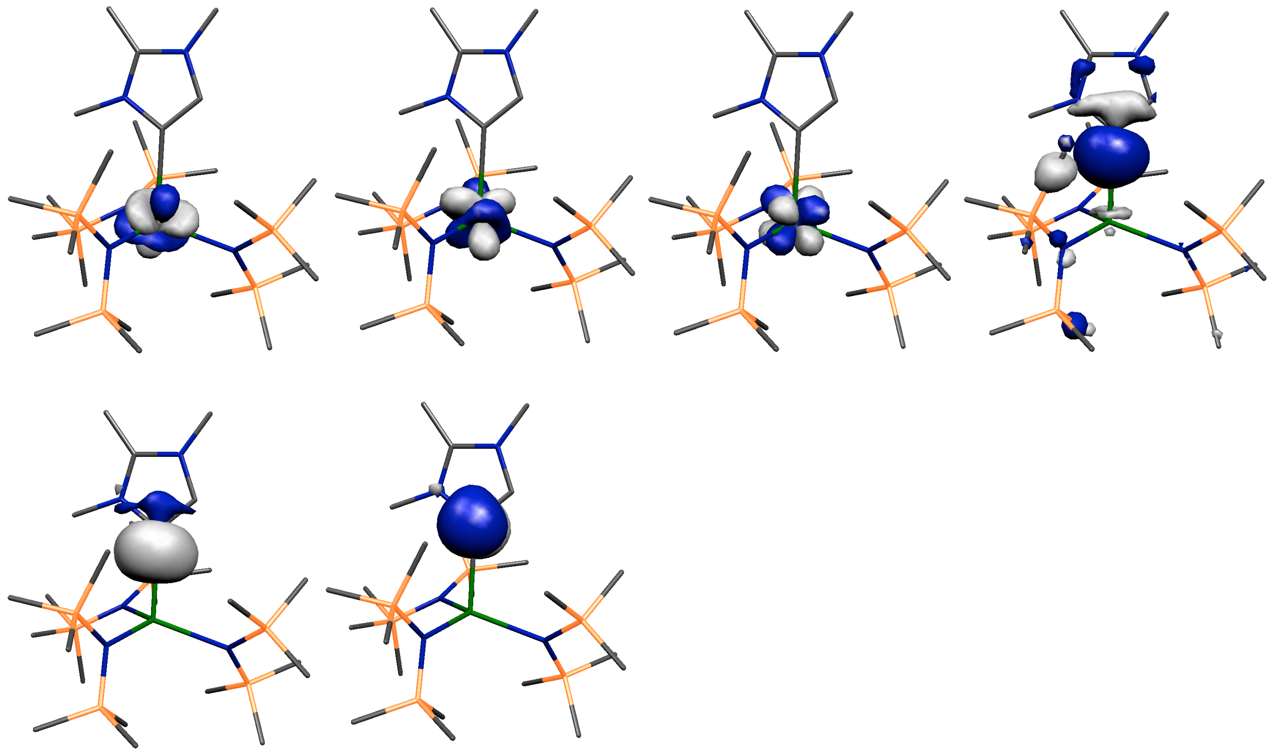

Figure S31. Left to right: HOMO (195a, −3.442 eV), HOMO−1 (194a, −3.484 eV), HOMO−2 (193a, −3.521 eV), HOMO−13 (182a, −6.440 eV), NBO of the Nd-C σ-bond, NBO of the π-symmetry acceptor orbital of the MIC for 3Nd. Hydrogen atoms are omitted for clarity.

Table S3. Final coordinates and single point energy of the geometry optimised structure of 3U.

1.C	2.505553	-0.723301	-4.731647
2.C	-0.380348	0.261754	-4.698893
3.C	0.059724	-3.473745	-3.994090
4.C	1.792720	1.666691	-3.090897
5.C	2.709842	-3.436682	-2.418482
6.C	-2.933352	-1.230570	-2.304334
7.C	-1.572390	2.801202	-1.652639
8.C	0.068740	-3.697498	-0.971698
9.C	-1.162236	5.613848	-0.519226
10.C	-5.098334	-0.211346	-0.510200
11.C	-0.486381	4.368713	-0.063050
12.C	3.522908	0.462978	-0.080677
13.C	-3.863236	-2.921309	0.077359
14.C	0.128758	2.151263	0.123663
15.C	0.795201	2.927868	1.052291
16.C	4.630515	-1.701730	1.701313
17.C	-3.543813	2.037166	1.738497
18.C	0.890996	5.350829	1.788415
19.C	2.220355	-4.055131	2.430151
20.C	-0.573910	-2.976629	2.719091
21.C	3.437139	0.787387	2.960565
22.C	-3.924951	-0.725166	3.006329
23.C	-1.286241	0.722580	3.221784
24.C	1.643533	-1.902847	4.504182
25.H	2.670314	-0.003914	-5.550995
26.H	-0.115776	0.928409	-5.536330
27.H	-0.771575	-0.674236	-5.125269
28.H	2.264242	-1.692017	-5.192121
29.H	0.487411	-3.064159	-4.920954
30.H	3.459868	-0.833466	-4.194686
31.H	0.159105	-4.570711	-4.042493
32.H	-1.205111	0.732191	-4.142187
33.H	-1.015120	-3.236282	-3.991327
34.H	1.823056	2.306554	-3.988849
35.H	3.284893	-3.072543	-3.281984
36.H	-3.612888	-1.910553	-2.844417
37.H	2.818713	1.599110	-2.698725
38.H	-1.253268	3.299259	-2.578242
39.H	-3.022201	-0.241682	-2.780136
40.H	2.737990	-4.538366	-2.439609
41.H	-1.905281	-1.578382	-2.478227
42.H	1.187535	2.182433	-2.330760
43.H	-1.550292	1.716674	-1.803121
44.H	-1.257544	5.625731	-1.613940
45.H	-2.599492	3.098091	-1.405884
46.H	3.228294	-3.106070	-1.505591
47.H	0.044120	-4.780174	-1.176486
48.H	-5.798469	-0.757556	-1.164382
49.H	3.485844	-0.211696	-0.948283
50.H	-0.976670	-3.387524	-0.814537

51.H	-4.986576	0.802233	-0.923360
52.H	-0.585893	6.500584	-0.225965
53.H	-4.668506	-3.338144	-0.549946
54.H	-2.176237	5.716878	-0.096064
55.H	0.618301	-3.570367	-0.027577
56.H	2.827359	1.295095	-0.273415
57.H	-2.999733	-3.599465	0.001292
58.H	4.534961	0.898655	-0.039567
59.H	-5.576917	-0.121963	0.474999
60.H	-4.428127	1.975557	1.088299
61.H	4.584026	-2.491109	0.936694
62.H	-4.206223	-2.945274	1.122281
63.H	5.584343	-1.164004	1.570706
64.H	1.415844	6.107250	1.190739
65.H	-2.834341	2.748608	1.286166
66.H	2.164583	-4.392855	1.384050
67.H	-0.869853	-3.479999	1.786730
68.H	1.521494	2.639855	1.801241
69.H	0.056456	5.821391	2.323828
70.H	-3.867428	2.459004	2.704696
71.H	4.662899	-2.189205	2.686632
72.H	-4.874591	-0.900099	2.480388
73.H	1.589235	4.935600	2.521202
74.H	4.300964	1.442224	2.759983
75.H	-0.611919	1.504000	2.843643
76.H	-1.280193	-2.155281	2.887247
77.H	1.794678	-4.855861	3.057602
78.H	3.284711	-3.961461	2.689136
79.H	2.551240	1.423038	3.111905
80.H	-3.497101	-1.708557	3.251495
81.H	-0.677159	-0.148843	3.491227
82.H	-0.712094	-3.704762	3.536019
83.H	3.622900	0.279708	3.919017
84.H	-4.163566	-0.220902	3.957277
85.H	-1.742966	1.103123	4.150997
86.H	2.718262	-1.722782	4.655138
87.H	1.108821	-0.983708	4.786728
88.H	1.342723	-2.695988	5.207967
89.N	0.769599	-1.080103	-2.220791
90.N	-0.662699	3.109574	-0.555547
91.N	-2.187793	-0.410459	0.503628
92.N	0.414787	4.259021	0.944156
93.N	1.595382	-1.187533	1.536435
94.Si	1.137524	-0.045639	-3.592144
95.Si	0.916272	-2.814577	-2.429397
96.Si	-3.441959	-1.154767	-0.476956
97.Si	3.194185	-0.468351	1.542296
98.Si	-2.705108	0.347057	2.008457
99.Si	1.249050	-2.443358	2.722849
100.U	0.083652	-0.401337	-0.080986

Energy: −532.45853645 eV

Table S4. Final coordinates and single point energy of the geometry optimised structure of 3Y.

1.C	0.335379	0.427502	-5.302519
2.C	3.090240	0.660738	-4.024963
3.C	1.249183	3.686680	-3.479155
4.C	1.361156	-1.781393	-3.491664
5.C	-1.643961	2.720441	-3.299628
6.C	-2.086815	-1.536782	-2.243913
7.C	-1.155378	-4.403212	-1.935033
8.C	-0.083742	3.413329	-0.783863
9.C	4.071617	2.261099	-0.351630
10.C	3.560126	-0.735490	-0.452890
11.C	2.092470	-4.324373	-0.182178
12.C	-3.012280	-3.154105	0.125372
13.C	-3.074122	1.184094	0.293029
14.C	-5.504658	1.495034	0.969037
15.C	-1.949374	0.547791	0.770050
16.C	-0.225046	-4.743785	1.715523
17.C	4.723564	0.456278	2.052629
18.C	-3.716438	0.252782	2.209241
19.C	1.740178	-2.574121	2.337365
20.C	2.682741	3.518251	2.725929
21.C	-0.179415	2.699369	2.895878
22.C	-1.580540	-0.844713	2.848834
23.C	-4.546203	-0.211560	3.355655
24.C	1.887863	1.003751	4.285497
25.H	0.785844	-0.159147	-6.120150
26.H	0.351957	1.483304	-5.611340
27.H	-0.717778	0.120301	-5.211977
28.H	3.500760	0.092558	-4.875964
29.H	1.360444	3.365410	-4.524784
30.H	3.164592	1.729997	-4.270292
31.H	-1.610198	2.452479	-4.366444
32.H	0.490257	-2.256366	-3.968116
33.H	2.259291	-2.170822	-3.997171
34.H	0.861110	4.718482	-3.490698
35.H	-2.032696	3.749647	-3.225193
36.H	3.741224	0.475000	-3.157896
37.H	2.253385	3.714936	-3.029943
38.H	-2.372218	2.042771	-2.825557
39.H	-1.361769	-1.357559	-3.047254
40.H	-3.005785	-1.934739	-2.705282
41.H	-0.304647	-4.294813	-2.623278
42.H	-2.045033	-4.653757	-2.536486
43.H	1.387669	-2.137259	-2.450922
44.H	-2.346593	-0.561346	-1.808078
45.H	-0.950495	-5.263947	-1.281871
46.H	3.406614	2.425886	-1.212582
47.H	2.849378	-0.754270	-1.293355
48.H	1.586699	-4.968062	-0.918385
49.H	4.556760	-0.755137	-0.920950
50.H	-0.409663	4.456042	-0.927239

51.H	5.083681	2.068515	-0.742850
52.H	2.753923	-3.646527	-0.742649
53.H	-3.213382	1.768658	-0.607327
54.H	-3.820196	-3.588677	-0.486855
55.H	0.875453	3.430895	-0.248177
56.H	-0.823045	2.940206	-0.118780
57.H	-5.502587	2.162182	0.102147
58.H	4.108644	3.193586	0.230301
59.H	3.448749	-1.672396	0.108642
60.H	2.727404	-4.970432	0.446227
61.H	-3.400558	-2.221031	0.566276
62.H	-6.195409	0.664118	0.776373
63.H	-0.785744	-5.368316	1.005439
64.H	-2.801123	-3.859460	0.942897
65.H	5.714773	0.280247	1.603394
66.H	-5.844839	2.060108	1.845061
67.H	2.483687	4.177564	1.867235
68.H	0.449135	-5.412715	2.276018
69.H	2.149343	-1.577801	2.118628
70.H	-0.470089	3.359000	2.065301
71.H	-0.945739	-4.330937	2.438388
72.H	4.456472	-0.439061	2.633848
73.H	2.572138	-3.219415	2.661344
74.H	4.827805	1.293667	2.757401
75.H	3.757452	3.288497	2.725309
76.H	-1.655761	-1.895591	2.548413
77.H	-0.541479	-0.523971	2.748108
78.H	-4.913150	-1.237769	3.195988
79.H	-0.971031	1.946142	3.016699
80.H	1.068958	-2.463396	3.204468
81.H	-5.416798	0.439323	3.499405
82.H	2.469645	4.095239	3.640520
83.H	-0.165937	3.308840	3.815001
84.H	-1.883508	-0.723254	3.894122
85.H	-3.965819	-0.206445	4.286886
86.H	2.921755	0.633920	4.347645
87.H	1.226774	0.126596	4.372236
88.H	1.705872	1.644844	5.163947
89.N	0.592126	0.917048	-2.297118
90.N	-0.101548	-2.216838	-0.041979
91.N	-4.143739	1.001939	1.167733
92.N	1.803910	0.986303	1.247917
93.N	-2.408848	-0.014674	1.977630
94.Si	1.297514	0.116364	-3.687643
95.Si	0.073116	2.572768	-2.482407
96.Si	-1.477535	-2.802299	-0.957884
97.Si	3.449945	0.794427	0.680153
98.Si	0.823671	-3.384961	0.877141
99.Si	1.566897	1.972134	2.675111
100.Y	0.328235	0.033965	-0.214373

Energy: −530.34781475 eV

Table S5. Final coordinates and single point energy of the geometry optimised structure of 3La.

1.C	0.801806	-0.686967	-4.554714
2.C	3.213926	-2.352339	-3.738604
3.C	-0.010281	-4.266812	-3.087584
4.C	-2.555273	-1.100029	-2.848483
5.C	2.911506	0.435353	-2.674413
6.C	-0.494144	2.640610	-2.323875
7.C	0.728383	5.490839	-1.875913
8.C	-4.810690	0.696517	-1.994235
9.C	2.209355	-4.438912	-0.975111
10.C	0.886117	4.276831	-1.026545
11.C	-4.404029	-1.942277	-0.546801
12.C	-0.598298	-3.672610	-0.152332
13.C	0.776796	2.128165	-0.212710
14.C	-3.314691	3.167993	0.144381
15.C	2.335442	5.338782	0.714489
16.C	1.568602	2.914531	0.596315
17.C	3.288650	-0.471275	0.868894
18.C	-4.609034	0.956111	1.785920
19.C	-1.759497	1.803899	2.354703
20.C	-2.042515	-2.052658	2.901087
21.C	3.436622	-2.467330	3.125911
22.C	2.880074	0.464927	3.743951
23.C	0.252040	-3.583152	4.115860
24.C	-0.326710	-0.777056	5.106853
25.H	1.406394	-0.304119	-5.393359
26.H	0.237857	-1.560326	-4.915913
27.H	3.742319	-1.887278	-4.587522
28.H	0.060393	0.084781	-4.294351
29.H	2.784452	-3.297931	-4.100054
30.H	0.668623	-4.181464	-3.949450
31.H	3.344026	0.834600	-3.606938
32.H	-3.209779	-1.775351	-3.422919
33.H	-0.973789	-3.823088	-3.381148
34.H	-2.285482	-0.266618	-3.515990
35.H	-0.175626	-5.341293	-2.904518
36.H	0.105485	2.213492	-3.137089
37.H	3.967811	-2.596323	-2.975100
38.H	0.078315	5.287262	-2.732279
39.H	-1.064060	3.497218	-2.696572
40.H	-5.446128	0.138601	-2.701777
41.H	1.701162	5.825260	-2.269231
42.H	-1.624546	-1.645283	-2.629728
43.H	-4.375108	1.545961	-2.542435
44.H	-1.198497	1.890990	-1.940317
45.H	2.315944	1.236239	-2.208568
46.H	3.744274	0.213843	-1.989397
47.H	2.995668	-4.466851	-1.743720
48.H	0.290277	6.325312	-1.307816
49.H	-5.471283	1.104505	-1.215437
50.H	-5.081504	-2.398988	-1.286965

51.H	1.923347	-5.480486	-0.754352
52.H	-4.043889	3.111945	-0.677317
53.H	-2.387976	3.612113	-0.253643
54.H	-1.544527	-3.133182	-0.324090
55.H	-0.870146	-4.738183	-0.100329
56.H	-3.723257	-2.730421	-0.189462
57.H	2.651663	-4.013076	-0.061098
58.H	3.029574	5.798714	-0.000060
59.H	3.135522	-1.239603	0.096153
60.H	-5.011784	-1.626080	0.314612
61.H	-3.715670	3.864831	0.898957
62.H	2.954151	0.490240	0.446606
63.H	-0.211195	-3.403642	0.844012
64.H	1.625165	6.095953	1.072188
65.H	4.375401	-0.378479	1.023456
66.H	-5.434717	0.808815	1.074349
67.H	2.906865	4.960037	1.567163
68.H	2.101970	2.663144	1.505201
69.H	-0.816545	2.249409	2.003928
70.H	-4.494172	0.023006	2.357430
71.H	-2.143300	-2.949672	2.270619
72.H	-4.920436	1.743328	2.492129
73.H	3.246449	-3.344915	2.489556
74.H	-2.429519	-1.201513	2.320640
75.H	-1.513849	0.910385	2.943798
76.H	4.519013	-2.259210	3.095543
77.H	-2.222249	2.526462	3.046353
78.H	0.396747	-4.274476	3.271656
79.H	-2.715329	-2.188463	3.763540
80.H	3.954437	0.711161	3.716864
81.H	2.310997	1.379299	3.511968
82.H	3.184255	-2.745983	4.159242
83.H	-0.541462	-4.002184	4.756726
84.H	1.181168	-3.578323	4.704268
85.H	2.630928	0.191649	4.780512
86.H	-0.630437	0.260167	4.899637
87.H	0.650975	-0.738252	5.611622
88.H	-1.049002	-1.201628	5.823179
89.La	-0.030836	-0.463338	0.105469
90.N	0.992663	-1.720714	-1.706297
91.N	0.370040	3.036892	-1.211943
92.N	-2.348954	0.279125	-0.187874
93.N	1.629920	4.215916	0.101963
94.N	0.764861	-1.175928	2.278446
95.Si	1.909138	-1.133806	-3.070469
96.Si	0.699133	-3.428480	-1.533945
97.Si	-3.467741	-0.471240	-1.306010
98.Si	-2.981193	1.462832	0.934639
99.Si	2.471303	-0.944410	2.523535
100.Si	-0.256467	-1.844260	3.530062

Energy: −530.83896896 eV

Table S6. Final coordinates and single point energy of the geometry optimised structure of 3Nd.

1.C	-1.759932	-0.897358	-4.919554
2.C	1.742630	-0.437534	-4.918113
3.C	-1.460692	2.111377	-4.524667
4.C	1.998285	2.184060	-3.400694
5.C	-3.163053	0.449093	-2.619590
6.C	0.522518	-4.610993	-2.060517
7.C	2.725263	-0.460472	-2.051891
8.C	-1.849245	-2.913665	-1.271036
9.C	-1.278298	3.106486	-0.598716
10.C	-4.044417	2.838148	0.546990
11.C	2.892737	-4.662752	0.589474
12.C	-0.912012	-5.156147	0.590350
13.C	-4.609869	-0.904545	0.931136
14.C	2.225731	2.308621	0.560205
15.C	1.448567	1.265212	1.009906
16.C	3.428679	-1.812279	1.311549
17.C	3.648856	4.174619	1.543920
18.C	-1.675967	3.299007	2.403136
19.C	-2.246607	-1.878650	2.650519
20.C	2.411102	2.382810	2.776357
21.C	1.489255	-3.479115	2.997181
22.C	-3.813945	0.563795	3.461386
23.C	0.915447	0.453786	3.325674
24.C	2.833607	2.796737	4.145365
25.H	-0.969850	-0.877970	-5.684137
26.H	1.155575	0.048135	-5.711365
27.H	-2.724659	-0.750856	-5.433372
28.H	-0.653387	2.134530	-5.272673
29.H	2.805874	-0.353504	-5.197384
30.H	-2.408014	2.284734	-5.060839
31.H	1.480047	-1.505689	-4.912871
32.H	-1.770230	-1.906593	-4.479783
33.H	1.472991	2.674818	-4.233834
34.H	3.080334	2.261968	-3.597278
35.H	-1.303605	2.959397	-3.841115
36.H	-3.987032	0.648771	-3.324323
37.H	1.768576	2.770996	-2.497740
38.H	0.847760	-3.868699	-2.804870
39.H	3.722211	-0.430577	-2.520489
40.H	-0.188552	-5.293241	-2.555179
41.H	-3.376625	-0.520088	-2.143978
42.H	-3.206862	1.216654	-1.836248
43.H	1.402639	-5.203292	-1.770932
44.H	-1.606091	-2.192522	-2.068305
45.H	2.483604	-1.516517	-1.857717
46.H	-1.735325	2.874951	-1.569039
47.H	2.823194	0.038375	-1.074452
48.H	-2.498638	-3.668305	-1.739796
49.H	-0.221968	2.810123	-0.649693
50.H	-1.302376	4.202133	-0.476789

51.H	3.323097	-4.524127	-0.413673
52.H	-4.071418	3.885618	0.200693
53.H	2.425428	2.643602	-0.451227
54.H	-2.470665	-2.412216	-0.512168
55.H	-4.520983	2.223821	-0.231758
56.H	-5.054497	-0.065334	0.374456
57.H	-1.601450	-5.830095	0.056438
58.H	-4.355845	-1.683471	0.194955
59.H	2.234902	-5.543278	0.554342
60.H	3.943610	-1.720678	0.343254
61.H	3.714631	4.469606	0.492634
62.H	-0.080903	-5.770222	0.968499
63.H	-4.669969	2.777003	1.450379
64.H	3.722669	-4.902814	1.274472
65.H	-5.388099	-1.319663	1.592990
66.H	-1.444963	-4.749903	1.462941
67.H	4.659346	3.963710	1.916910
68.H	3.105794	-0.800622	1.600378
69.H	-1.598870	-2.406265	1.933931
70.H	4.170087	-2.156974	2.051321
71.H	3.213677	5.004152	2.115319
72.H	-1.897915	4.372540	2.284174
73.H	-0.589581	3.200073	2.560368
74.H	-4.410787	1.447925	3.195887
75.H	-3.001172	-2.598353	3.006505
76.H	0.842661	-4.369585	3.026582
77.H	-2.185797	2.952898	3.314410
78.H	-1.617876	-1.617081	3.516376
79.H	1.339388	-0.551912	3.229340
80.H	2.367914	-3.683079	3.631091
81.H	0.917518	-2.662266	3.464090
82.H	-4.480024	-0.127755	4.003385
83.H	-0.145785	0.434164	3.049020
84.H	-3.030295	0.885995	4.163564
85.H	3.900469	3.060228	4.155847
86.H	2.265896	3.670513	4.502110
87.H	1.018432	0.793067	4.359738
88.H	2.689344	1.983227	4.863585
89.N	-0.140543	0.179428	-2.559964
90.N	0.673449	-2.557812	0.222908
91.N	-1.963592	0.605742	0.996416
92.N	2.800972	2.988661	1.632086
93.N	1.598472	1.362841	2.408326
94.Nd	-0.203915	-0.384221	-0.266242
95.Si	-1.529764	0.449833	-3.592588
96.Si	1.463801	0.360331	-3.215905
97.Si	-0.296938	-3.773455	-0.564670
98.Si	-2.230808	2.331725	0.856375
99.Si	2.018393	-3.084253	1.207363
100.Si	-3.099405	-0.333299	1.937319

Energy: −531.52863205 eV

CASSCF-SO *ab initio* Calculations

General

In order to confirm the trivalent formulation of **3U** and corroborate the EPR spectroscopic and SQUID magnetometric data, CASSCF-SO calculations on the individual disordered conformers of **3U** were performed using MOLCAS 8.0.[15] We employed the ANO-RCC-VTZP basis for uranium, ANO-RCC-VDZP for the first coordination sphere donor atoms and the ANO-RCC-VDZ basis for the rest of the atoms,[16,17] using the geometry from X-ray crystallography with no optimisation. The active space in all cases was three electrons in the seven 5f orbitals located on uranium; attempts to enlarge the active space to include the ligand orbitals using the RAS-probing method failed,[18] instead resulting in the inclusion of the uranium 6p and 6d orbitals (for all disordered isomers). All 35 quartets and 112 doublets of the $5f^3$ configuration were considered in the state-averaged CASSCF orbital optimisations, as well as the subsequent spin-orbit mixing. Cholesky decomposition of the two electron integrals was performed with a threshold of 10^{-8} to save disk space and reduce computational demands. We have also performed calculations on the published crystal structure of **1U** in order to compare its properties. Using the same approach as given above, we find a distinctly different electronic structure where the *g*-values for the ground doublet reflect the three-fold axial symmetry, and the 3rd and 4th excited doublets are substantially higher in energy, Table S11.

Table S7. Summary of selected properties from CASSCF-SO calculations on 3U.

Disorder fraction	g_x	g_y	g_z	1st excited state	μ_{eff} @ 2 K (μ_B)	μ_{eff} @ 298 K (μ_B)
0.475	0.69	2.5	4.2	206 cm^{-1}	2.46	3.28
0.391	0.72	2.2	4.4	238 cm^{-1}	2.50	3.27
0.135	0.69	2.5	4.1	238 cm^{-1}	2.44	3.27
**Weighted average**	**0.70**	**2.4**	**4.3**	**223 cm^{-1}**	**2.48**	**3.28**

Table S8. Electronic states from CASSCF-SO calculations on 3U (0.475 disorder fraction).

Energy (cm^{-1})	g_x	g_y	g_z
0	0.69	2.5	4.2
206	0.26	1.0	2.5
391	0.52	1.9	3.3
950	0.24	0.4	4.5
1118	0.78	2.8	3.9

Table S9. Electronic states from CASSCF-SO calculations on 3U (0.391 disorder fraction).

Energy (cm^{-1})	g_x	g_y	g_z
0	0.72	2.2	4.4
238	0.72	1.2	2.4
381	0.28	2.4	2.9
955	0.21	0.9	4.2
1150	0.75	2.4	4.4

Table S10. Electronic states from CASSCF-SO calculations on 3U (0.135 disorder fraction).

Energy (cm^{-1})	g_x	g_y	g_z
0	0.69	2.5	4.1
238	0.33	1.0	2.2
414	0.28	2.1	3.2
963	0.06	0.7	4.2
1158	0.80	2.4	4.3

Table S11. Electronic states from CASSCF-SO calculations on 1U.

Energy (cm^{-1})	$g_{\parallel}$	$g_{\perp}$
0	0.62	3.3
221	1.9	0.25
439	0.78	2.3
1354	2.2	2.6
1630	5.8	0.03

References

1. J. L. Stewart, R. A. Andersen, *Polyhedron* **1998**, *17*, 953.
2. A. Fürstner, M. Alcarazo, R. Goddard, C. W. Lehmann, *Angew. Chem. Int. Ed.* **2008**, *47*, 3210.
3. S. Fortier, J. L. Brown, N. Kaltsoyannis, G. Wu, T. W. Hayton, *Inorg. Chem.* **2012**, *51*, 1625.
4. P. B. Hitchcock, M. F. Lappert, L. Maron, A. V. Protchenko, *Angew. Chem. Int. Ed.* **2008**, *47*, 1488.
5. C. Fonseca Guerra, J. G. Snijders, G. te Velde E. J. Baerends, *Theor. Chem. Acc.* **1998**, *99*, 391.
6. G. te Velde, F. M. Bickelhaupt, S. J. A. van Gisbergen, C. Fonseca Guerra, E. J. Baerends, J. G. Snijders T. Ziegler, *J. Comput. Chem.* **2001**, *22*, 931.
7. S. H. Vosko, L. Wilk, M. Nusair, *Can. J. Phys.* **1980**, *58*, 1200.
8. A. D. Becke, *Phys. Rev. A.* **1988**, *38*, 3098.
9. J. P. Perdew, *Phys. Rev. B.* **1986**, *33*, 8822.
10. S. Portmann, H. P. Luthi, *Chimia* **2000**, *54*, 766.
11. NBO 5.0: E. D. Glendening, J. K. Badenhoop, A. E. Reed, J. E. Carpenter, J. A. Bohmann, C. M. Morales, F. Weinhold, (Theoretical Chemistry Institute, University of Wisconsin, Madison, WI, 2001); http://www.chem.wisc.edu/~nbo5.
12. R. F. W. Bader, *Atoms in Molecules: A Quantum Theory*, Oxford University Press, New York, 1990.
13. R. F. W. Bader, *J. Phys. Chem. A.* **1998**, *102*, 7314.
14. http://www.quimica.urv.es/XAIM.
15. F. Aquilante, J. Autschbach, R. K. Carlson, L. F. Chibotaru, M. G. Delcey, L. De Vico, I. F. Galván, N. Ferré, L. M. Frutos, L. Gagliardi, M. Garavelli, A. Giussani, C. E. Hoyer, G. L. Manni, H. Lischka, D. Ma, P. -Å. Malmqvist, T. Müller, A. Nenov, M. Olivucci, T. B. Pedersen, D. Peng, F. Plasser, B. Pritchard, M. Reiher, I. Rivalta, I. Schapiro, J. Segarra-

Martí, M. Stenrup, D. G. Truhlar, L. Ungur, A. Valentini, S. Vancoillie, V. Veryazov, V. P. Vysotskiy, O. Weingart, F. Zapata, R. Lindh, J. Comp. Chem. **2016**, *37*, 506.

16. B. O. Roos, R. Lindh, P.-Å. Malmqvist, V. Veryazov, P.-O. Widmark, *J. Phys. Chem. A* **2004**, *108*, 2851.
17. B. O. Roos, R. Lindh, P.-Å. Malmqvist, V. Veryazov, P.-O. Widmark, *Chem. Phys. Lett.* **2005**, *409*, 295.
18. V. Veryazov, P. Å. Malmqvist, B. O. Roos, *Int. J. Quant. Chem*. **2011**, *111*, 3329.
